# Mechano-regulated cell–cell signaling in the context of cardiovascular tissue engineering

**DOI:** 10.1007/s10237-021-01521-w

**Published:** 2021-10-06

**Authors:** Cansu Karakaya, Jordy G. M. van Asten, Tommaso Ristori, Cecilia M. Sahlgren, Sandra Loerakker

**Affiliations:** 1 Department of Biomedical Engineering, Eindhoven University of Technology, Eindhoven, the Netherlands; 2 Institute for Complex Molecular Systems, Eindhoven University of Technology, Eindhoven, the Netherlands; 3 Department of Biomedical Engineering, Boston University, Boston, MA USA; 4 Faculty of Science and Engineering, Biosciences, Åbo Akademi, Turku, Finland

**Keywords:** Cell–cell signaling, Mechano-regulation, Growth and remodeling, Tissue organization, Mechanobiology, Computational modeling

## Abstract

Cardiovascular tissue engineering (CVTE) aims to create living tissues, with the ability to grow and remodel, as replacements for diseased blood vessels and heart valves. Despite promising results, the (long-term) functionality of these engineered tissues still needs improvement to reach broad clinical application. The functionality of native tissues is ensured by their specific mechanical properties directly arising from tissue organization. We therefore hypothesize that establishing a native-like tissue organization is vital to overcome the limitations of current CVTE approaches. To achieve this aim, a better understanding of the growth and remodeling (G&R) mechanisms of cardiovascular tissues is necessary. Cells are the main mediators of tissue G&R, and their behavior is strongly influenced by both mechanical stimuli and cell–cell signaling. An increasing number of signaling pathways has also been identified as mechanosensitive. As such, they may have a key underlying role in regulating the G&R of tissues in response to mechanical stimuli. A more detailed understanding of mechano-regulated cell–cell signaling may thus be crucial to advance CVTE, as it could inspire new methods to control tissue G&R and improve the organization and functionality of engineered tissues, thereby accelerating clinical translation. In this review, we discuss the organization and biomechanics of native cardiovascular tissues; recent CVTE studies emphasizing the obtained engineered tissue organization; and the interplay between mechanical stimuli, cell behavior, and cell–cell signaling. In addition, we review past contributions of computational models in understanding and predicting mechano-regulated tissue G&R and cell–cell signaling to highlight their potential role in future CVTE strategies.

## Introduction

Cardiovascular diseases are one of the leading causes of morbidity and mortality worldwide and represent a major economic and social burden to society due to healthcare expenditures and productivity losses (Timmis et al. 2020; Virani et al. 2020). Many cardiovascular diseases, such as coronary artery disease or calcified aortic valve disease, often require surgical interventions to replace or repair blood vessels or heart valves. The current blood vessel replacement options, namely autologous vessels and synthetic vascular grafts, have several limitations. Autologous vessels (e.g., saphenous veins) have limited availability and poor functionality in patients with systemic vascular diseases (Harskamp et al. 2013; Hess et al. 2014; McNichols et al. 2021). Synthetic grafts are often associated with the occurrence of thrombosis and poor patency rate, particularly for small-diameter vessels (Eslami et al. 2001; Haruguchi and Teraoka 2003; Sarkar et al. 2006; Pashneh-Tala et al. 2016). Current replacement options for diseased heart valves can be classified as mechanical or bioprosthetic, and are associated with several drawbacks as well. Mechanical valves are susceptible to thromboembolic complications and require life-long anticoagulation treatment (Zilla et al. 2008; Lim et al. 2017). Bioprosthetic valves are prone to structural degeneration, which is generally associated with additional valve replacements, especially for young patients (Welke et al. 2011; Head et al. 2017). Most importantly, none of these blood vessel and heart valve replacements are able to grow or remodel to accommodate changing conditions and functional requirements. This is a great limitation especially for pediatric patients, who inevitably outgrow their replacement and therefore require multiple reoperations.

Cardiovascular tissue engineering (CVTE) can potentially overcome the limitations of current replacements. This field aims to create living replacements that can grow, repair, remodel and thereby provide lifetime functionality (Langer and Vacanti 1993). In the classical CVTE paradigm, cells are isolated from the patient and seeded onto a scaffold material within a bioreactor, to form a native-like tissue that is then implanted into the patient. More recently, also other approaches have been proposed that bypass the in vitro cell culture phase, and solely rely on the regenerative capacity of the body to induce neotissue formation directly at the functional site (Lee et al. 2014; Wissing et al. 2017). Due to the presence of living cells, such engineered tissues have the intrinsic ability to grow and adapt in response to changing demands.

Despite some promising examples (Sutherland et al. 2005; Hoerstrup et al. 2006; McAllister et al. 2009; Hibino et al. 2010; Talacua et al. 2015), there is still a need for improvement because the capacity of tissue-engineered blood vessels (TEBVs) and heart valves (TEHVs) to grow and adapt to changing circumstances is still largely unknown, and the remodeling processes after the implantation are still poorly understood. In addition, TEBVs and TEHVs do not always exhibit proper long-term functionality. The main functional problems of these tissues after implantation are, for example, related to stenosis, thrombus formation, and calcification (Gottlieb et al. 2010; Schmidt et al. 2010; Tara et al. 2015; Yang et al. 2016; Sugiura et al. 2016). In addition, suboptimal mechanical properties of TEHVs may lead to the progressive development of valve insufficiency or even tissue rupture (Flanagan et al. 2009; Weber et al. 2013; Syedain et al. 2015; Reimer et al. 2017). The function of cardiovascular tissues is strongly correlated with their biomechanical properties (Fung 1993; Nerem 2000; Sacks et al. 2009), which are in turn directly determined by their (physiological) tissue organization. Therefore, we hypothesize that establishing a native-like tissue organization is necessary for overcoming the limitations of current CVTE constructs, which can only be achieved in a controllable manner when the growth and remodeling (G&R) mechanisms of (engineered) cardiovascular tissues are adequately understood.

Cells are the main drivers of G&R. Cell behavior is intrinsically determined by cell–cell signaling pathways and can be manipulated via external factors such as mechanical cues (Bukoreshtliev et al. 2013; Han et al. 2018). For example, mechanical cues strongly affect cell differentiation, proliferation, apoptosis, and matrix synthesis, all processes that are strongly related to tissue G&R (Humphrey 2006). However, the underlying biological mechanisms are still scarcely elucidated. Recent studies have highlighted an increasing number of mechanoresponsive characteristics of cell–cell signaling pathways (Hiepen et al. 2020; Stassen et al. 2020). Therefore, mechano-mediated cell–cell signaling could explain the link between mechanical cues and cell behavior that determines G&R. Fully unraveling this interplay between mechanics and cell–cell signaling could open new possibilities to control cellular behavior in CVTE, with the purpose to induce functional G&R, by tuning both mechanical cues and cell–cell signaling pathways. Our review focuses on cell–cell signaling in vascular and valvular cells and does not consider other signaling phenomena, such as those involving inflammatory cells. In short, an enhanced understanding of mechano-regulated cell–cell signaling and the resulting increased ability to control cell behavior and tissue G&R may be utilized to optimize CVTE and improve the functional organization of engineered tissues.

Within this review, we will highlight the potential key role of mechano-mediated cell–cell signaling pathways in CVTE and the future directions in this field. In particular, we will first discuss the relationship between tissue organization, biomechanics, and function of native vessels and heart valves. Thereafter, we will discuss the obtained tissue organization in previous CVTE studies, together with their limitations. Next, we will focus on the interplay between cellular behavior, mechanical cues, and cell–cell signaling to identify the potential role of mechano-regulation of signaling pathways in G&R of TEBVs and TEHVs. Throughout the review, we will also emphasize the past and future contributions of computational models in advancing understanding and predicting tissue G&R, cell–cell signaling, and CVTE strategies. Finally, we will conclude with a description of the challenges that remain to be addressed in CVTE and present an outlook on future directions. We focus on both blood vessels and heart valves because they are similar from a tissue engineering perspective, as they share many of the methods and techniques adopted in this field, as well as many of the limitations and challenges we still face. In addition, the G&R of both these tissues are affected by mechanical stimuli and cell–cell signaling pathways and the computational models we discuss can generally be applied to both tissues.

## The organization and structure of cardiovascular tissues

### Tri-laminar structure in native blood vessels

**Fig. 1:**
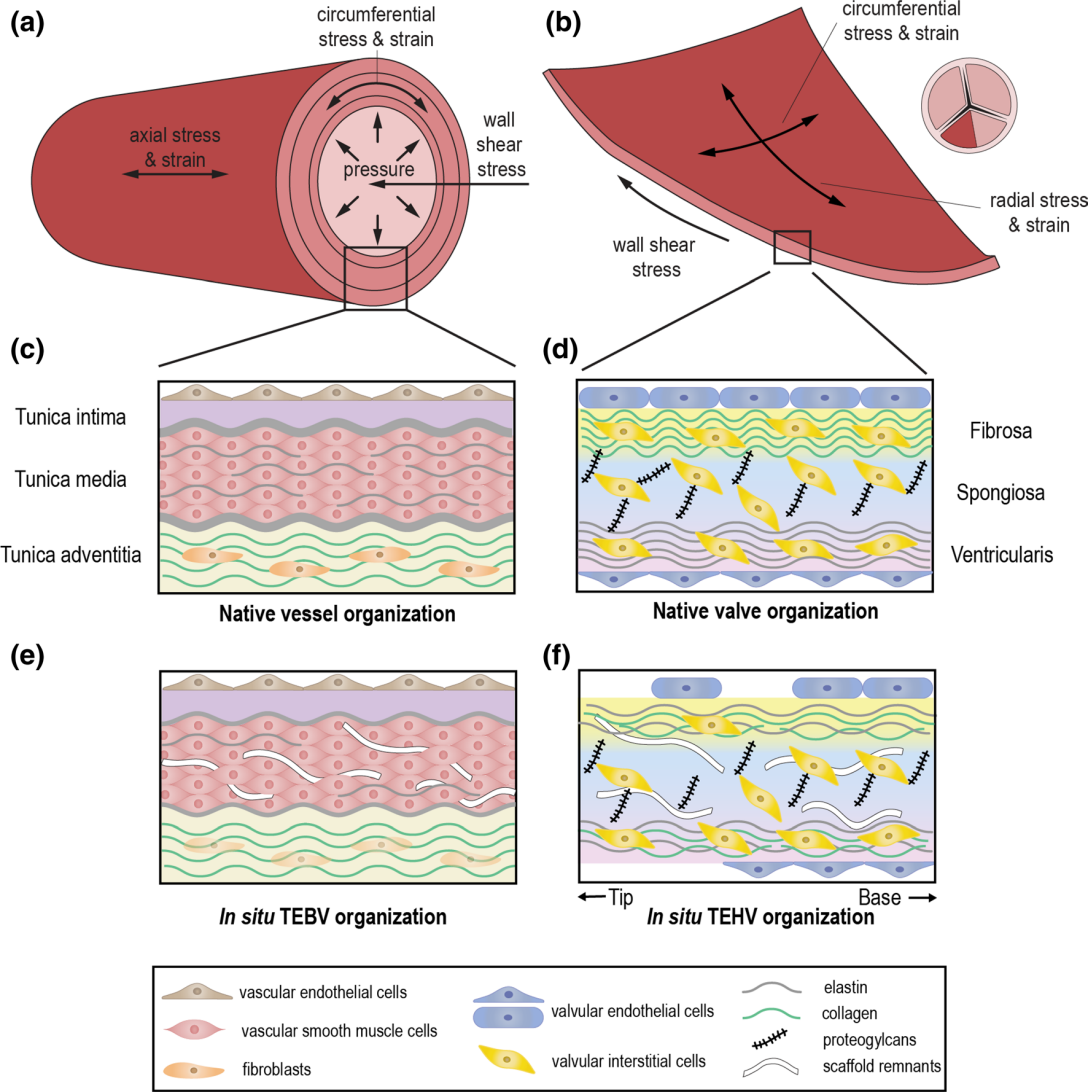
The mechanical loads on blood vessels and heart valves, and the schematic representation of the tissue organization **a** the mechanical loads on arterial blood vessels **b** the mechanical loads on the semilunar heart valve leaflets. A 2D top view of a closed heart valve is shown with half of one of the leaflets highlighted, representing the portion of the leaflet which is visualized in the main 3D illustration. **c** tissue organization of native arterial blood vessels **d** tissue organization of native semilunar heart valves **e** organization of in situ TEBVs **f** organization of in situ TEHVs

The vasculature is comprised of a network of blood vessels, going from arteries to veins, where each type has a specific structure and function. Arteries enable the transport of blood away from the heart toward arterioles and capillaries, where the chemical and metabolic exchange between blood and tissues occurs. In turn, blood is transported back to the heart through venules and then larger veins. In terms of general structure, both arteries and veins are composed of three layers, known as tunica intima, tunica media, and tunica adventitia (Fig. 1c). Each of them exhibits unique structural and functional features.

The tunica intima is the inner layer of blood vessels. This layer is composed of a single layer of endothelial cells (ECs), lining the vascular wall, and a basal lamina. Large arteries also present a subendothelial area, between the basal lamina and internal elastic lamina, which separates the tunica intima from the tunica media. ECs in the inner arterial layer are oriented along the axis of the vessel wall, which corresponds to the direction of blood flow (Langille and Adamson 1981). These cells act as the first barrier separating blood from the surrounding tissue. As such, ECs play crucial roles in various cardiovascular processes, including vasculogenesis, angiogenesis, coagulation, and inflammation (Cines et al. 1998). Moreover, in response to mechanical cues (Awolesi et al. 1995; Topper et al. 1996), ECs secrete mediators to control vasoconstriction (Yanagisawa et al. 1988) and vasodilation (Furchgott and Zawadzki 1980), which are crucial processes for regulating blood pressure and flow. ECs also produce the components of the basal lamina to which they adhere. The basal lamina supports the (EC) layer and acts as selectively permeable barrier to regulate the passage of molecules between tissue layers (Arends and Lieleg 2016). It is mainly composed of laminin, collagen IV, perlecan, and nidogen (Fox et al. 1991; Battaglia et al. 1992; Hopf et al. 1999). Similarly, ECs also produce the components of the subendothelial area that lies between the basal and elastic laminae, which contains microfibrils and collagen fibers (Gerrity and Cliff 1972; Davis 1993) and serves as an anchor for the ECs to the elastic lamina. In humans, this area also contains intimal smooth muscle cells (Schwartz et al. 1995). It is not yet clear if these cells are trapped in this area during development or if they have a specific key function, such as in the development of atherosclerosis.

The tunica media is the middle layer of blood vessels. It is composed of several layers of vascular smooth muscle cells (VSMCs), elastin sheets (lamellae), a network of elastic fibers, collagen fibers, and layers of several other extracellular matrix (ECM) proteins. In large arteries, the media is separated from the adventitia by an external elastic lamina. VSMCs are responsible for the production and the organization of ECM in the media layer. Enclosed within elastin lamellae and surrounded by collagen fibers and proteoglycan-rich ECM, VSMCs are aligned in the direction of collagen fiber bundles (Dingemans et al. 2000; O’Connell et al. 2008), which are oriented in the tunica media at an angle of approximately 30° with respect to the circumferential direction (Holzapfel 2006). The elastin lamellae are protruded by thin elastin fibers, to which the VSMCs adhere. VSMCs play important roles in blood vessel function, development, and homeostasis. These cells maintain the vascular tone through cell contraction and relaxation. Furthermore, VSMCs generally express a differentiated quiescent phenotype in healthy homeostatic vessels, and they can change phenotype towards a migratory and proliferative state upon biological and mechanical stimuli (Owens et al. 2004). This ability to switch phenotypes is key to regulate the G&R of the vessel wall.

Finally, the tunica adventitia is the outermost layer of the vessel wall. It consists of a collagen-rich ECM and a variety of cell types including fibroblasts, progenitor cells, and immunomodulatory cells (Stenmark et al. 2013). The adventitia gives stability and strength to the vessel and connects the vessels to the surrounding tissues. It also provides nutrients and oxygen to the cells in the vessel wall, and it enables the removal of waste products through a network of small vessels called vasa vasorum (Wolinsky and Glagov 1967). Fibroblasts are the most abundant cell type in the adventitia. These cells produce the adventitial ECM and remodel the ECM in response to stress or injury (Stenmark et al. 2013). Collagen type I and type III constitute a major part of ECM in the adventitia (Howard and Macarak 1989). The main role of these collagen fibers, which are mainly axially aligned in the adventitia (Holzapfel 2006) and present a highly nonlinear stiffness, is to prevent the vessel wall from rupture at high blood pressures.

### Tri-laminar structure in native heart valves

The main function of heart valves is to maintain the unidirectionality of blood flow during the cardiac cycle. The atrioventricular mitral and tricuspid valves allow blood flow from the atria to the ventricles in diastole and prevent backflow from the ventricles during systole. The semilunar aortic and pulmonary valves separate the ventricles from the aorta and pulmonary artery, respectively, and open during systole to allow blood flow from the ventricles to the arteries. The architecture and localized distribution of the ECM are crucial for the physiological function of the heart valves. In general, the belly region of semilunar heart valves has a trilaminar structure composed of an organized ECM and interspersed valvular interstitial cells (VICs), covered by a monolayer of valvular endothelial cells (VECs) on both sides of the leaflets (Schoen 2008) (Fig. 1d). The commissures where the leaflets come together have a monolayer and fibrous structure (Misfeld and Sievers 2007). The first two layers in the belly region in heart valves are known as the fibrosa and spongiosa. The third layer is called ventricularis in semilunar valves and atrialis in atrioventricular valves. The atrioventricular valves exhibit both a ventricularis on the ventricular side and an atrialis on the atrial side (Gross and Kugel 1931; Sacks et al. 2019).

The fibrosa is located close to the outflow surface of the valves. It is mainly composed of circumferentially oriented fibrillar collagen (Latif et al. 2005; Ayoub et al. 2018). This dense network of collagen fibers provides strength to the valve (Sauren et al. 1980; Kodigepalli et al. 2020). The ventricularis layer of semilunar valves and the atrialis layer of atrioventricular valves face the inflow side and are mainly composed of radially oriented elastic fibers that facilitate tissue movement by providing extension and subsequently recoil of the valve tissue (Vesely 1998). The spongiosa is the middle layer and mainly contains glycosaminoglycans (GAGs) and proteoglycans which interconnect the collagen and elastin fibers. This layer is histologically distinct, but if it is a functionally distinct layer is still under debate (Eckert et al. 2013; Buchanan and Sacks 2014).

The two main cell populations in heart valves are VICs and VECs. VICs are embedded through all three layers of the leaflets, while VECs line the surfaces of the valves. VICs are responsible for matrix maintenance, synthesis, and remodeling (Latif et al. 2005). They are highly plastic and can express various phenotypes upon injury or alterations in mechanical state. In mature heart valves, VICs are mainly quiescent to maintain the physiological valve function and homeostasis (Aikawa et al. 2006) and can be activated for valve remodeling (Liu et al. 2007a). VECs also have important roles in physiological functioning of the valves. For example, VECs regulate platelet adhesion and coagulation, act as a functional barrier between the blood and the valve tissue, and interact with the VICs to regulate their phenotypes (Butcher and Nerem 2007). VECs have different gene expression profiles and obtain different morphologies on different sides of the valves; they are elongated and flattened on the ventricular side and cuboidal on the arterial side of the semilunar valves. This difference in shape is related to the presence of high- and low-shear forces, respectively (Maron and Hutchins 1974).

### Similarities and differences in tri-laminar structure of vessels and valves

Blood vessels and heart valves both have a trilaminar architecture and layer-specific ECM organization which are crucial for ensuring proper functionality. Both tissues contain one (predominantly) fibrous layer, the tunica adventitia and the fibrosa, which provide functional strength. The cells in the inner layer of both tissues, VSMCs and VICs, are quiescent and nonproliferative in the homeostatic state and can alter their phenotype to regulate vascular and valvular remodeling, respectively. However, the expression of α-smooth muscle actin (α-SMA) and myosin heavy chain is elevated in quiescent VSMCs, whereas these proteins are markers of activated VICs (Liu et al. 2007a). Important to note, however, is that even though VICs do have some contractile properties, they are more fibroblast-like cells, compared to VSMCs (Filip et al. 1986; Latif et al. 2015). Each organizational layer of blood vessels includes different cell types with different functions, whereas the VICs are interspersed throughout all layers of the valves, and VECs cover the blood-contacting surfaces of the valves. In addition, the morphology of ECs and VECs is different based on their differential response to shear stress (Butcher et al. 2004). ECs align parallel to the flow, while VECs align perpendicular to flow. These differences in composition are due to the different functions of vascular and valvular tissues.

### Cardiovascular tissue biomechanics and functional organization

Each component and organizational feature of native blood vessels and heart valves contributes to ensuring proper tissue functionality under hemodynamic loading conditions. In this section, we discuss the specific hemodynamic loads acting on blood vessels and heart valves, the resulting mechanical stimuli experienced by each tissue, and the functional organization adopted by each tissue to optimally accommodate these mechanical stimuli.

#### Blood vessel biomechanics

Blood flowing through the vessels exerts a frictional force acting on the inside of the vessel, which is called wall shear stress (Fig. 1a). Blood flow also exerts pulsating pressure onto the blood vessels, causing them to dilate which results in cyclic stress and strain in the vascular wall, both in the circumferential and the axial directions (Fig. 1a). The high elasticity and extensibility of elastin (Davis 1995) in the media layer allow the blood vessel to expand during systole, reducing the resistance to blood flow, and recoil during diastole, maintaining a pressure gradient required to drive the blood through the rest of the vasculature (Humphrey 2002; Cocciolone et al. 2018). Meanwhile, the much stiffer collagen fibers give the vessel strength and resilience to protect against excessive strains. The helical organization of collagen fibers in the media layer (Sect. 2.1) ensures that the vessel can withstand loads in both circumferential and axial directions (Holzapfel et al. 2000). The collagen fibers in the adventitia are initially coiled and only become elastically stretched and start bearing load at high pressures. Under these circumstances, their relatively high stiffness becomes dominant in the tissue mechanical response. This way, the collagen fibers act as a protective sheath that allows normal dilation of the wall but prevents over-dilation and rupture (Holzapfel et al. 2000; Humphrey 2002). In addition to blood pressure, active contraction of smooth muscle cells, mediating vasoconstriction and vasodilation, also contributes to the circumferential stress and strain in the vascular wall. Moreover, residual stress (and pre-stretch) is present within the tissue even when external loads have been removed. This residual stress is hypothesized to arise during development as a result of elastin fibers being extended due to somatic growth after the fibers have reached maturity (Davis 1995; Cardamone et al. 2009). Moreover, collagen fibers incorporated into the tissue at a preferred deposition stretch might also contribute to the establishment of residual stresses (Humphrey and Rajagopal 2002; Cardamone et al. 2009). From a functional point of view, it has been suggested that residual stress ensures the uniformity of strain and/or stress throughout the vessel wall (Fung 1991; Destrade et al. 2012) and thereby facilitates the establishment of mechanical homeostasis.

#### Heart valve biomechanics

A similar correlation between components and mechanical function can be observed in native heart valves, which are similarly subjected to mechanical loads due to the cyclic expulsion of blood from the heart. In particular, heart valve leaflets are subjected to wall shear stress throughout the entire cardiac cycle; bending as a result of the opening and closing of the leaflets; and pressure when the leaflets are closed (Fig. 1b). As a result of bending and pressure, heart valve leaflets experience stress and strain in both the circumferential and radial directions (Fig. 1b). During bending, stresses and strains are heterogeneous across the leaflet layers (Sacks et al. 2009). The high flexibility of radially organized elastin fibers in the ventricularis enables the leaflets to open and close by bending easily and provides the main restorative force (Vesely 1997). Collagen fibers in the ventricularis become more recruited upon closing of the leaflets, resulting in a more compliant tissue in the opened state to allow large extensions and a stiffer tissue in the closed state to limit further extension (Vesely and Noseworthy 1992; Vesely 1997). Thus, they exhibit a similar protective role as in the adventitia layer of blood vessel. In the closed configuration, circumferential collagen fibers in the fibrosa provide the valves with the tensile strength required to resist the blood pressure (Sacks et al. 2009; Ayoub et al. 2016). The high stiffness of these collagen fibers limits deformations when the valves are closed to maintain coaptation (Schoen and Levy 1999; Sacks et al. 2009). Taken together, the combined organization and alignment of collagen and elastin make the valve leaflets very pliable in the unloaded state, enabling efficient opening and closing, and very stiff in the loaded state, ensuring proper valve closure. Finally, the main function of the components in the spongiosa appears to be absorbing shear stress to enable the ventricularis and fibrosa to move relative to each other during bending and pressurization (Schoen and Levy 1999; Sacks et al. 2009).

In conclusion, the organization of blood vessels and heart valves is tightly linked with the mechanical loads that they experience. Both the layered structure and alignment of fibers in these tissues ensure proper tissue function under physiological loading conditions. In particular, collagen fibers are the main load-bearing components and essential for providing strength, while elastin is crucial for ensuring flexibility. Therefore, inducing a proper tissue organization and adequate distribution and alignment of elastin and collagen fibers within engineered cardiovascular tissues is of paramount importance for the functionality of these tissues. In addition, cellular infiltration and cell-mediated G&R are critical to ensure a proper ECM turnover and adaptive capabilities of the tissue.

## Growth and remodeling in cardiovascular tissue engineering

### In vitro cardiovascular tissue engineering

In vitro CVTE aims at creating functional cardiovascular tissues outside the body that can be subsequently implanted to replace diseased or malformed cardiovascular tissues. When following this in vitro approach, (preferentially autologous) cells are seeded onto a scaffold and afterward subjected to biochemical and mechanical stimuli within a bioreactor in order to induce tissue formation prior to implantation (Langer and Vacanti 1993). Many strategies have been proposed to construct functional tissues in vitro*.* Most of them can be broadly categorized based on the used scaffold type, such as synthetic materials (e.g., polyglycolide, poly-L-lactide, poly(ester-urethane)urea), natural materials (e.g., fibrin, collagen), and decellularized biological matrices (Pashneh-Tala et al. 2016; Goins et al. 2019), although some strategies do not require scaffolds but only supports (e.g., in the case of sheet-based tissue engineering) (L’Heureux et al. 1998). In this section, we discuss the final tissue organization, in terms of cellular and ECM distribution, obtained with in vitro cardiovascular tissue engineering approaches.

#### Organization in in vitro tissue-engineered blood vessels

##### Decellularized native matrices

Decellularized native matrices have often been used as scaffold material for the in vitro creation of TEBVs, as they immediately provide an optimal ECM organization and corresponding mechanical properties. In this approach, vascular tissue is harvested from an allogeneic or xenogeneic donor and decellularized by using biological agents, chemical agents, or physical methods (Crapo et al. 2011). Autologous cells are then seeded onto the preserved ECM. The tissue construct is subsequently cultured in vitro and, ultimately, implanted into the host. Animal studies have shown that this approach can yield confluent EC layers in the intima of the engineered vessels, surrounded by layers of VSMCs in the media (Kaushal et al. 2001; Cho et al. 2005; Tillman et al. 2012). Since the decellularization process generally preserves collagen fibers, the internal and external elastin lamina, and the dense elastic layers in the media, the trilaminar organization of native blood vessels is generally present also in these engineered tissues. In agreement with the concept that organization corresponds to function, clinical studies following this strategy demonstrated promising functionality of such constructs implanted as portal veins of pediatric patients (Olausson et al. 2012, 2014). However, the scarce availability of donor homografts and risks of zoonotic infections from xenografts present substantial limitations in terms of the potential for large-scale clinical translation.

##### Synthetic and natural scaffolds

Polymeric scaffolds with a synthetic material represent a valid alternative to decellularized matrices, as they in principle have an unlimited availability and they still offer the possibility to control scaffold properties toward native-like features. The commonly used synthetic polymers for vascular tissue engineering applications include polyglycolide (PGA), poly-L-lactide (PLLA), and poly(ester-urethane)urea (PEUU) (Niklason et al. 1999; Shinoka et al. 2005; Hoerstrup et al. 2006; Nieponice et al. 2010). In vitro TEBVs using synthetic scaffolds to substitute large arteries in the low-pressure circulation, e.g., the pulmonary artery or inferior vena cava, usually exhibit a cellular organization with a luminal EC layer and medial VSMC layers similar to native tissues (Shinoka et al. 1998; Watanabe et al. 2001; Hoerstrup et al. 2006; Cummings et al. 2012). Moreover, there are no observed scaffold traces. In addition, elastic fibers have been observed in the medial layer (Shinoka et al. 1998; Watanabe et al. 2001; Hoerstrup et al. 2006; Cummings et al. 2012), although sometimes lower in content compared to native levels (Hoerstrup et al. 2006; Cummings et al. 2012). On the other hand, the collagen content and structure in these constructs is similar to native tissues (Shinoka et al. 1998; Watanabe et al. 2001; Hoerstrup et al. 2006). The native-like cell and ECM structure in these studies correspond to adequate functionality. Pre-clinical studies with large-diameter vessel implantations have demonstrated promising functionality of the engineered blood vessels up to 80–100-week follow-up (Hoerstrup et al. 2006; Cummings et al. 2012). Clinical studies with pediatric patients have also demonstrated that grafts are mostly patent 10 years after implantation (Shinoka et al. 2005; Hibino et al. 2010; Shoji and Shinoka 2018). Despite these encouraging results, it should be noted that these engineered vessels had a large diameter. As such, they were less susceptible to neointimal hyperplasia and thrombus formation compared to small-diameter grafts, and they were implanted in a relatively low-pressure circulation. Thus, translating this success into small-diameter and high-pressure vessels is a challenge that still needs to be overcome (Mirensky et al. 2009).

Polymeric synthetic and natural scaffold-based approaches of small-diameter constructs are mostly successful in achieving a native-like cellular organization, but they cannot yet obtain a native-like ECM organization. In general*, *in vivo studies have shown that the luminal surface of these TEBVs is always covered by an EC monolayer (Niklason et al. 1999; Swartz et al. 2005; Liu et al. 2007b; Iwasaki et al. 2008; He et al. 2010; Koch et al. 2010; Nieponice et al. 2010; Soletti et al. 2010). This may not be surprising, since ECs are generally seeded onto the lumen of the tubular constructs already during the in vitro stage (Niklason et al. 1999; Swartz et al. 2005; Liu et al. 2007b; Koch et al. 2010). Dynamic preconditioning in vitro also helps to achieve a layered VSMC organization in the tunica media, with a circumferential orientation of VSMCs (Niklason et al. 1999; Seliktar et al. 2000; Iwasaki et al. 2008; He et al. 2010; Koch et al. 2010; Nieponice et al. 2010; Schutte et al. 2010). In vitro culture also promotes collagen formation (Niklason et al. 1999; Swartz et al. 2005; Buttafoco et al. 2006; Liu et al. 2007b; Iwasaki et al. 2008; He et al. 2010; Koch et al. 2010; Nieponice et al. 2010; Soletti et al. 2010). Circumferentially aligned collagen fibers have been observed in vitro and in vivo, although not predominantly localized in the outer layers of engineered vessels (Swartz et al. 2005; Iwasaki et al. 2008; He et al. 2010; Nieponice et al. 2010). However, a native-like elastin content and the establishment of cohesive elastin sheet formation in the middle layer have not been achieved yet (Swartz et al. 2005; Koch et al. 2010), despite attempts with multi-layered synthetic scaffolds that slightly improved elastin deposition and organization (Iwasaki et al. 2008; He et al. 2010). Therefore, these constructs should be improved especially in terms of elastin content and elastin organization.

##### Cell-sheet approach

Another method to create in vitro TEBVs consists of forming tubular grafts without using a scaffold or a supporting matrix. The rationale of this approach is to solely use cultured cells to construct TEBVs without the addition of a scaffolding material (L’Heureux et al. 1998). In this approach, cell sheets corresponding to the different vessel layers are rolled around a mandrel, which is removed after tissue maturation. Sheet-based tissue engineering has been adopted in preclinical and clinical studies with high success rates (L’Heureux et al. 1998, 2006; McAllister et al. 2009). The preclinical studies have shown that, when such grafts are implanted as arterial replacements, they exhibit the native-like trilayer organization in terms of cells and ECM. Furthermore, their burst pressure is comparable with the native vessels, due to presence of a similar ECM organization (L’Heureux et al. 1998, 2006). In agreement with their native-like composition, it has been shown that the grafts can be functional and patent in the long-term (L’Heureux et al. 2006). The clinical studies have shown that this method can achieve good functionality after up to 20 months, even when patients have underlying pathologies (McAllister et al. 2009; Wystrychowski et al. 2014). Overall, these studies show that establishing a native-like tissue organization is correlated with good long-term functionality. A major drawback of the overall approach, however, is that the in vitro culture procedure is often time-consuming, expensive, and logistically challenging.

#### Organization in in vitro tissue-engineered heart valves

Compared to in vitro vascular tissue engineering, attempts at establishing a native-like tissue organization in in vitro tissue-engineered heart valves have been less successful so far.

##### Decellularized native matrices

Similar to vessels, decellularization of allogeneic or xenogeneic tissues, combined with seeding of autologous cells, has been adopted to tissue engineer heart valves in vitro. With this approach, valve tissue is formed in vitro, which is then implanted into the host (Boccafoschi et al. 2015). Decellularized native matrices seeded with cells already have a native-like ECM organization at the start of the in vitro culture period (Schenke-Layland et al. 2003; Cushing et al. 2005; Kim et al. 2006; Lichtenberg et al. 2006; Dohmen et al. 2007). This approach in fact preserves the collagen and elastin content of the valve leaflets and thus the trilaminar organization of the valves. However, active myofibroblast-like cells have been observed throughout the thickness of the leaflets in the explants, as well as incomplete endothelialization and thickening of the leaflets (Steinhoff et al. 2000; Kim et al. 2006). These outcomes could be related to the type of cells seeded and the use of relatively short follow-up times. Despite these imperfections, clinical studies using decellularized allografts and xenografts seeded with autologous cells have shown good hemodynamic performance and functionality (Cebotari et al. 2006; Dohmen et al. 2007, 2011). Still, the availability of allografts is limited, and zoonotic risks of xenografts are still present. In addition, there is an uncertainty regarding the G&R capacity of TEHVs from decellularized native matrices because of the limited potential for cell infiltration (Weber et al. 2013).

##### Synthetic and natural scaffolds

To avoid the risk of xenogeneic diseases and limited supply of allografts, biodegradable synthetic and natural scaffolds are used as an alternative (Fioretta et al. 2018). This approach has shown that ECs generally cover the leaflet surfaces of TEHVs (Hoerstrup et al. 2000; Sodian et al. 2000a, 2010; Sutherland et al. 2005; Flanagan et al. 2009), although an incomplete EC layer has also been reported in vitro and in vivo (Flanagan et al. 2007; Schmidt et al. 2010). Cells found in the internal layers of the leaflet are often *α*-SMA positive, as opposed to the cells in healthy native leaflets (Hoerstrup et al. 2000, 2002; Flanagan et al. 2007, 2009; Schmidt et al. 2010; Sodian et al. 2010; Weber et al. 2011, 2012). On the other hand, Sutherland et al*.* have reported that *α*-SMA-positive cells have been detected throughout the leaflets at the time of tissue implantation, and these cells have localized at the subendothelial layer, similar to native leaflets, after 8 months in vivo (Sutherland et al. 2005). This observation could be associated with the use of longer follow-up times compared to other studies.

Dynamic loading increases the tissue formation and ECM remodeling in TEHVs (Engelmayr et al. 2006; Eckert et al. 2011; D’Amore et al. 2016). Particularly, the combination of cyclic flexure and laminar flow, which better mimics the physiological mechanical conditions, accelerates the tissue formation compared to applying either cyclic flexure or laminar flow (Engelmayr et al. 2006). Moreover, ECM production is promoted by cyclic strain up to a certain threshold (30%) from which a decrease in production is observed with a further increase in strain up to 50% (D’Amore et al. 2016). However, the total amount of ECM produced by cells in TEHV is often less compared to the ECM content in native counterparts (Shinoka et al. 1995; Breuer et al. 1996; Hoerstrup et al. 2002; Sodian et al. 2010). In addition, there are also still several challenges with respect to mimicking the native ECM organization. In particular, collagen is often deposited in both the outer layers of engineered leaflets instead of only the fibrosa layer (Sodian et al. 2000b, a; Neidert and Tranquillo 2006; Flanagan et al. 2007, 2009; Schmidt et al. 2010; Weber et al. 2011). Furthermore, despite their general abundance, GAGs are not always localized in the middle layer (Sodian et al. 2000a; Flanagan et al. 2007). Finally, elastin has only rarely been observed (Sutherland et al. 2005; Sodian et al. 2010) and usually has not been synthesized in vitro or in vivo studies using polymeric scaffolds (Sodian et al. 2000b, a; Stock et al. 2000; Hoerstrup et al. 2002; Neidert and Tranquillo 2006; Flanagan et al. 2007, 2009; Schmidt et al. 2010; Weber et al. 2011).

The lack of a native-like organization often leads to a gradual loss of functionality after implantation. The main complications that have resulted in TEHV failure are leaflet shortening, regurgitation, and stenosis (Stock et al. 2000; Sodian et al. 2000a; Gottlieb et al. 2010; Schmidt et al. 2010; Weber et al. 2011). In addition, even though the scaffold changes substantially from its initial state (Eckert et al. 2011), remnants of synthetic scaffold material have still been detected in some studies after explantation of engineered tissues (Sodian et al. 2000a; Schmidt et al. 2010), which creates a potential risk of calcification (Sugiura et al. 2017). Natural scaffolds, on the other hand, have often provided insufficient mechanical strength (Neidert and Tranquillo 2006; Flanagan et al. 2007, 2009) and are generally subjected to high degrees of compaction.

#### General limitations of in vitro cardiovascular tissue engineering

The main limitations of in vitro CVTE are the length, costs, and logistic challenges associated with the in vitro culture procedure (L’Heureux et al. 1998; Tremblay et al. 2014). Upscaling the production of in vitro engineered tissues to meet clinical demands is another significant challenge that is difficult to resolve (Niklason and Lawson 2020). Finally, in vitro engineered cardiovascular tissues often lack a native-like tissue organization which strongly correlates with reduced tissue functionality. Alternative tissue engineering approaches not relying on in vitro cell culture have been recently introduced to overcome (part of) these challenges, as outlined in the next section.

### In situ cardiovascular tissue engineering

To overcome the limitations of in vitro tissue engineering approaches discussed in the previous section, much attention has recently been shifted toward the in situ tissue engineering approach. In situ tissue engineering is defined as the regeneration of tissues from a readily available scaffold that is implanted directly at the functional site in the body (Mol et al. 2009; Wissing et al. 2017; Bouten et al. 2018). This method relies on the presence of a resorbable scaffold temporarily taking over the tissue function, while host cells repopulate the scaffold and form new autologous tissue (Mol et al. 2009; Roh et al. 2010; Wissing et al. 2017; Bouten et al. 2018). The choice of scaffold material is of utmost importance. It may either consist of synthetic polymers (Khosravi et al. 2015; Kluin et al. 2017) or be obtained by decellularizing a xenograft, allograft, or in vitro tissue-engineered matrix (TEM) (Dijkman et al. 2012; Goecke et al. 2018; Wolkers and Hilfiker 2021). To ensure immediate availability, these decellularized scaffolds can be procured ahead of time and safely stored for long periods of time (Dijkman et al. 2012; Goecke et al. 2018; Wolkers and Hilfiker 2021). Scaffolds are usually acellular before implantation, although they may also be pre-seeded right before surgery (“on-the-fly”) (Hibino et al. 2011; Harrington et al. 2011). The decellularization process aims to reduce the immunogenicity of the decellularized xenografts, allografts, and TEMs (Goldstein et al. 2000; da Costa et al. 2005; Dijkman et al. 2012). Nevertheless, an immunological response is sometimes still observed due to incomplete decellularization or the residual presence of active inflammatory stimuli, especially in xenografts (Simon et al. 2003; Kasimir et al. 2006; Filippo et al. 2013). Synthetic scaffolds present the additional advantage of being highly tailorable in terms of material and microstructural properties, providing better reproducibility than grafts cultured in vitro (Breuer et al. 2004; Capulli et al. 2016; Fioretta et al. 2020). Finally, in situ tissue engineering does not require patient-specific cells in contrast to in vitro methods, as TEMs can be cultured using readily available cell sources (Reimer et al. 2017; Emmert et al. 2018; Motta et al. 2019). Based on our definition of in situ tissue engineering, autografts fall outside the scope of the current review. Interested readers are referred to the following recent review articles on this topic: (Mazine et al. 2018; Nappi et al. 2020a, 2020b). To provide a current overview of the tissue organization that is achieved in in situ engineered tissues, we discuss some recent results of studies highlighting the composition and organization of in situ TEBVs and TEHVs. In particular, we focus our attention on recellularization, matrix synthesis, and the structure and distribution of cells and matrix components in the neotissue. Tables 1 and 2 give an overview of the characteristics of these studies and their main findings in terms of tissue organization.

**Table 1:** Overview of the context and outcome of a selection of recent studies on in situ TEBVs, with an emphasis on tissue organization

References	Methodology	Follow-up (weeks)	Model system	Cellularization	ECM components	Structure	Functionality
Syedain et al. (2016)	Decellularized allograft	50	Lamb pulmonary artery	Complete endothelial layer Extensive repopulation by mature VSMCs, along entire graft length	Collagen deposition higher than native (180%) Elastin deposition lower than native (46%)	Elongated VSMCs in circumferential direction Collagen aligned in circumferential direction	Normal physiological function No major complications
Koobatian et al. (2016)	Decellularized xenograft	13	Sheep carotid artery	Confluent endothelial layer Cell infiltration, approaching native density No mature smooth muscle	Collagen content similar to native No fibrillar elastin Variable elastin content between explants	Circumferentially aligned VSMCs	Patent grafts No major complications Mechanical properties similar to or higher than native Incomplete remodeling
Row et al. (2015)	Decellularized xenograft (pre-seeded with ECs and VSMCs)	13	Sheep left common carotid artery interposition	Confluent endothelial layer, aligned in direction of flow Cell density similar to native	Collagen observed	Circumferentially aligned VSMCs Native fibrillar organization not reached	Patent grafts Diameter of grafts typically higher than native
Kirkton et al. (2019)	Decellularized TEM	200	Human haemodialysis access	Some variability in endothelial coverage Increasing repopulation with VSMCs and/or myofibroblasts		Stratification into adventitial and medial layers observed Elongated and circumferentially aligned VSMCs	
Khosravi et al. (2015)	Synthetic graft	104	Mouse inferior vena cava		Deposition of collagen fibers (both collagen I and collagen III)		Patent grafts No major complications Variability in mechanical properties, some compliance mismatch
Sugiura et al. (2016)	Synthetic graft (bi-layered)	8	Mouse infrarenal aortic interposition	Endothelial layer observed Cell infiltration and layer of VSMCs observed	Deposition of both collagen and elastin observed		Acute thrombosis in 2 grafts 1 mouse died of undetermined cause Other grafts patent and without major complications
Sugiura et al. (2017)	Synthetic graft (bi-layered)	8	Mouse infrarenal aortic interposition	Endothelial layer observed Cell infiltration and layer of VSCMs observed Significantly more cells in fast degrading scaffolds compared to slow degrading)			Calcification observed in slow degrading scaffolds (none in fast degrading)
Zhu et al. (2015)	Synthetic graft (bi-layered)	12	Rat abdominal aorta	Full endothelial layer, mature cobble-stone like ECs Mature and contractile VSMCs observed	Collagen observed, less compact than native Elastin deposition progressed slowly	ECs aligned in direction of flow Circumferentially aligned VSMCs Circumferentially aligned collagen	Vascular function observed, contraction less than native No major complications Evidence found for signaling between VSCMs and ECs
Yang et al. (2016)	Synthetic graft (bi-layered)	52	Rat abdominal aorta	Confluent endothelial layer, spindle-shaped ECs VSMCs observed Cellularization similar to native (except in densest scaffold sheath)	Elastin and collagen deposition similar to native (except in densest scaffold sheath: more collagen, less elastin)	Tri-laminar structure observed Intermediate density scaffold sheath showed best VSMC alignment Circumferentially aligned elastin	Patency approx. 80–86% Some thrombosis in all groups Graft rupture in lowest density scaffold sheaths Calcification in highest density scaffold sheaths
Talacua et al. (2015)	Synthetic graft (with shields to prevent transanastomotic cell infiltration)	13	Rat abdominal aorta	Confluent endothelium with elastic lamina Medial layer with VSMCs	Collagen fibers observed	Circumferentially aligned collagen Collagen fiber bundles lining endothelium and outside, more loose fibers in the center	Patent grafts No major complications
Khosravi et al. (2016)	Synthetic graft (bi-layered)	52	Mouse infrarenal aortic interposition	Cell infiltration of VSMCs and myofibroblasts observed Contractile VSMCs observed	Substantial collagen and elastin deposition	Elastin organization initially lamellar, later lost	Patent grafts Calcification present No other major complications Grafts stiffer than native
Tara et al. (2015)	Synthetic graft	52	Mouse infrarenal aortic interposition	Confluent endothelial layer VSMCs observed	Gradual increase of collagen, higher than native Elastin observed, less than native		12 mice died of graft rupture Aneurysm and dilation in most grafts Normal blood flow and patency Progressive and ongoing remodeling

**Table 2:** Overview of the context and outcome of a selection of recent studies on in situ TEHVs, with an emphasis on tissue organization

References	Methodology	Follow-up (weeks)	Model system	Cellularization	ECM components	Structure	Functionality
Theodoridis et al. (2015)	Decellularized allograft	52	Sheep pulmonary valve	Spatial variability in cellularization	Collagen observed Collagen content correlated with cellular repopulation	Repopulation more extensive on ventricular than arterial side Poor repopulation in tip Collagen on both ventricular and arterial sides of leaflet Dense collagen in lamina fibrosa No cells in lamina fibrosa	Some calcification No major complications
Tudorache et al. (2016)	Decellularized allograft	87	Sheep aortic & pulmonary valve	Incomplete cellularization and endothelial coverage More cells positive for α-SMA than in native valves	Collagen deposition observed	Stronger repopulation on ventricular side than arterial side No cells in lamina fibrosa Collagen in lamina fibrosa GAGs in lamina spongiosa	Some minor stenosis, retraction, insufficiency and pinpoint thrombosis
Miller et al. (2016)	Decellularized allograft	26	Pig pulmonary valve	Incomplete endothelium		Endothelial coverage only at base of leaflet Incomplete remodeling, initiating at base of leaflets	Significant stenosis in all valves Moderate regurgitation which worsened in all valves 1 case of calcification
Goecke et al. (2018)	Decellularized allograft	26	Sheep pulmonary valve	Incomplete endothelial coverage VICs observed in leaflets		Repopulation stronger on ventricular than pulmonary side More repopulation on proximal side, decreasing toward tip No cells in lamina fibrosa Tri-layered structure maintained Collagen on both sides of leaflet	Satisfactory function in most valves Regurgitation in 2 valves Small thrombus in 2 valves Significant deterioration in 1 valve
Zafar et al. (2015)	Decellularized xenograft	34	Sheep tricuspid valve	Complete endothelial layer Dense cellularization Cells transitioning from remodeling phenotype to homeostasis		Approximation of organized ECM stratification on proximal side of leaflet Lack of ECM organization on distal side of leaflet	One case of severe regurgitation No other major complications Mechanical properties similar to native
Hennessy et al. (2017)	Decellularized xenograft	22	Sheep pulmonary valve	Cellular infiltration observed (remodeling phenotype) VEC monolayer formation	ECM remained intact Signs of collagen deposition	Incomplete endothelium on pulmonary side Cellularization predominant on ventricular side and infiltrated toward pulmonary side	Normal functioning valves Moderate insufficiency due to leaflet retraction Mechanical properties similar to native
Van Rijswijk et al. (2020)	Decellularized xenograft	26	Sheep pulmonary valve	Limited endothelial coverage Cellularization highly variable and lower than native levels	No elastin deposition observed	No signs of remodeling into tri-layered structure	7 of 20 animals died Moderate to severe stenosis Severe regurgitation Signs of calcification
Syedain et al. (2015)	Decellularized TEM	24	Sheep Aortic valve	Endothelium and basement membrane observed Cellularization observed, but DNA content lower than native	Collagen deposition observed but content lower than native Elastin detected in some valves	Cellularization and endothelialization extensive at base and lower belly, but partial toward tip ECM components localized with cellularization	Normal heart function No major complications Unchanged mechanical properties
Reimer et al. (2017)	Decellularized TEM	22	Sheep pulmonary valve	Incomplete cellularization and endothelial coverage	Collagen and elastin deposition Collagen content higher than native, elastin content lower than native No mature elastin observed	Fairly uniform distribution of cells Collagen and elastin on both sides of leaflets	3 premature endings Increasing regurgitation to moderate/severe Leaflet shortening Higher mechanical properties than native
Emmert et al. (2018)	Decellularized TEM (with geometric constraints)	52	Sheep pulmonary valve	Homogeneous endothelial coverage with typical EC morphology Substantial cell infiltration, but lower than native Phenotype cells in leaflets similar to native	Collagen deposition Dense, homogenous, and wavy collagen matrix Elastin present No change in GAG content	Tri-layered structure generally not observed Collagen alignment observed, but less than native Cell repopulation observed throughout leaflet	Generally good functionality without major complications Signs of functional remodeling
Motta et al. (2018)	Decellularized TEM	16	Sheep pulmonary valve	Confluent endothelium DNA content higher than native Moderate expression of α-SMA-positive cells	Collagen content similar to native GAG content less than native Traces of elastin	Densely packed collagen fibers Cells predominantly in wall and hinge regions Elastin on ventricular side	Good acute performance Progressive severity of regurgitation No stenosis or calcification
Kluin et al. (2017)	Synthetic scaffold	52	Sheep pulmonary valve	Confluent endothelium on pulmonary side, near-confluent on ventricular side Extensive colonization by host cells, DNA content similar to native	Collagen content similar to native Sparse elastic fibers	Collagen and elastin on same side of the leaflet Elastic fibers mainly on pulmonary side	Well functioning valves One case of severe regurgitation No other major complications Good mechanical integrity
Fioretta et al. (2020)	Synthetic scaffold (pre-seeded with bone marrow mononuclear cells or unseeded)	24	Sheep pulmonary valve	Incomplete endothelium Cellularization observed	Collagen formation observed Elastic fibers observed	Endothelium absent toward the tip	Variability in organization, cell infiltration and ECM deposition between leaflets Pathological calcification, regurgitation, leaflet fusion, and maladaptive remodeling in pre-seeded scaffolds

#### Organization in in situ tissue-engineered blood vessels

##### Cellular repopulation

A key aspect in achieving a functional neotissue is the realization of proper repopulation of the scaffold with host cells. Cells infiltrate the scaffolds gradually (Tara et al. 2015; Koobatian et al. 2016; Khosravi et al. 2016; Kirkton et al. 2019), typically from the transmural and transanastomotic sides in rodent models (Talacua et al. 2015). However, it has been shown that infiltration from the circulation is also possible (Row et al. 2015; Talacua et al. 2015). This may be particularly useful for engineering longer vascular grafts in humans, where transanastomotic infiltration is rare (Talacua et al. 2015). Many studies indeed report the presence of host cells in explanted grafts. For example, they are generally well populated by ECs, resulting in the formation of an extensive endothelial layer (Fig. 1e) (Zhu et al. 2015; Tara et al. 2015; Talacua et al. 2015; Koobatian et al. 2016; Yang et al. 2016; Syedain et al. 2016; Marosfoi et al. 2017; Kirkton et al. 2019) which has been shown to develop progressively over time (Zhu et al. 2015; Koobatian et al. 2016; Marosfoi et al. 2017). The newly formed endothelium is often similar to native tissue, with aligned ECs (Zhu et al. 2015; Koobatian et al. 2016; Yang et al. 2016) and a cobble-stone cell shape (Zhu et al. 2015). In addition to ECs, the presence of VSMCs is also very common in in situ TEBVs (Fig. 1e) (Row et al. 2015; Zhu et al. 2015; Tara et al. 2015; Talacua et al. 2015; Koobatian et al. 2016; Yang et al. 2016; Khosravi et al. 2016; Sugiura et al. 2016, 2017). These VSMCs often display a mature, contractile phenotype (Zhu et al. 2015; Yang et al. 2016; Khosravi et al. 2016; Syedain et al. 2016; Kirkton et al. 2019). This mature phenotype seems to develop gradually over time, and several studies have shown a phenotypic switch taking place from undifferentiated, synthetic VSMCs to differentiated, contractile VSMCs (Zhu et al. 2015; Kirkton et al. 2019). Interestingly, very few studies seem to report on the presence of fibroblasts, which are present in the adventitia layer of native blood vessels. This aspect deserves more attention due to the role of fibroblasts in ECM production and remodeling (Sect. 2.1).

##### ECM formation

The infiltrating cells have displayed the ability to deposit collagen and elastin fibers in both synthetic and decellularized scaffolds (Fig. 1e) (Row et al. 2015; Zhu et al. 2015; Tara et al. 2015; Talacua et al. 2015; Koobatian et al. 2016; Yang et al. 2016; Khosravi et al. 2016; Sugiura et al. 2016; Syedain et al. 2016). This deposition of matrix components may be influenced by a variety of factors. For example, pre-seeding decellularized xenografts with VSMCs enhanced collagen deposition in a sheep model (Row et al. 2015), while cyclic stretching increased elastin production in a mouse model (Tara et al. 2015), confirming the importance of mechanical stimuli in regulating tissue G&R. The collagen content in TEBVs is often extensive and similar to or higher than native levels (Tara et al. 2015; Koobatian et al. 2016; Khosravi et al. 2016; Syedain et al. 2016). The elastin content, on the other hand, is generally lower than or similar to that of native vessels (Fig. 1e) (Tara et al. 2015; Yang et al. 2016; Syedain et al. 2016), indicating that achieving proper elastin deposition might be more challenging. This may be partly explained by the fact that elastin turnover in adults is much slower than collagen turnover (Langille 1993; Cocciolone et al. 2018), so adult host cells may not be able to produce elastin at the same rate as collagen. Finally, it is worth noting that our current understanding of tissue organization in TEHVs is limited as many studies do not take this aspect into consideration. This presents an additional complication in the search for improved functionality of TEHVs.

##### Functionality and complications

As may be expected from the generally favorable cellular repopulation and abundant presence of matrix components, recent studies on in situ TEBVs have generally been successful, resulting in patent and functional grafts (Khosravi et al. 2015, 2016; Row et al. 2015; Tara et al. 2015; Talacua et al. 2015; Koobatian et al. 2016; Yang et al. 2016; Sugiura et al. 2016). However, some complications still occasionally occur, such as aneurysm formation (Tara et al. 2015), graft rupture (Tara et al. 2015; Yang et al. 2016), thrombosis (Yang et al. 2016; Sugiura et al. 2016), and calcification (Yang et al. 2016; Khosravi et al. 2016; Sugiura et al. 2017). Some of these issues emphasize the key importance of an appropriate scaffold degradation profile, as scaffolds degrading too slowly have been associated with calcification (Khosravi et al. 2016; Sugiura et al. 2017), and scaffold breakdown occurring too quickly can result in aneurysms and graft rupture if it is not balanced by sufficient neotissue formation (Tara et al. 2015).

Consistent with the generally favorable functionality, recent in situ TEBVs have presented many signs of advanced organization resembling that of native vessels. For example, a tri-laminar organization has been shown in both a pre-clinical murine model (Yang et al. 2016) and in a clinical setting (Kirkton et al. 2019). In addition, a medial layer with organized VSMCs has been observed in several studies (Row et al. 2015; Talacua et al. 2015; Yang et al. 2016; Kirkton et al. 2019) as well as the presence of circumferentially aligned VSMCs (Row et al. 2015; Zhu et al. 2015; Koobatian et al. 2016; Syedain et al. 2016; Kirkton et al. 2019). A circumferential alignment has also been observed for collagen fibers (Zhu et al. 2015; Talacua et al. 2015) and elastin fibers (Yang et al. 2016), which is similar to the native situation in the media layer.

#### Organization in in situ tissue-engineered heart valves

##### Cellular repopulation

Repopulation of in situ TEHVs generally seems less extensive and more variable compared to in situ TEBVs (Fig. 1f). For example, varying degrees of endothelial layer formation have been observed in TEHV leaflets. A few studies reported an extensive or complete endothelial layer (Zafar et al. 2015; Syedain et al. 2015; Emmert et al. 2018; Motta et al. 2018), but unfortunately moderate endothelialization is more common (Tudorache et al. 2016; Miller et al. 2016; Reimer et al. 2017; Hennessy et al. 2017; Goecke et al. 2018; Fioretta et al. 2020; van Rijswijk et al. 2020). Interestingly, limited endothelial coverage is seen mostly toward the tips of the leaflets, while the base is more extensively populated (Fig. 1f) (Syedain et al. 2015; Miller et al. 2016; Reimer et al. 2017; Fioretta et al. 2020). This suggests that the formation of endothelial layers starts from the leaflet base, closer to the native tissue, and progresses toward the tip. This theory is supported by the observation that endothelial coverage seems to improve over time, both when comparing multiple time points within the same studies (Zafar et al. 2015; Kluin et al. 2017; van Rijswijk et al. 2020), and when comparing endothelialization across studies with different follow-up times (Theodoridis et al. 2015; Zafar et al. 2015; Miller et al. 2016; Reimer et al. 2017; Hennessy et al. 2017; Emmert et al. 2018; Goecke et al. 2018; Fioretta et al. 2020; van Rijswijk et al. 2020). However, complete endothelialization has been reported as early as after 16 weeks (Motta et al. 2018), while an incomplete coverage has been observed even after 20 months (Tudorache et al. 2016), thereby demonstrating that strong variability in outcomes can occur and further understanding and improvement are therefore needed.

Repopulation of TEHVs with other cell types is similarly variable and different degrees have been observed, from limited and partial repopulation (Theodoridis et al. 2015; Tudorache et al. 2016; Miller et al. 2016; Reimer et al. 2017; Hennessy et al. 2017; Coyan et al. 2019; van Rijswijk et al. 2020) to extensive repopulation (Zafar et al. 2015; Syedain et al. 2015; Kluin et al. 2017; Emmert et al. 2018; Goecke et al. 2018; Fioretta et al. 2020). Nevertheless, apart from rare exceptions (Kluin et al. 2017), the DNA content of TEHVs is often still lower than that of native valves (Syedain et al. 2015; Emmert et al. 2018). Despite signs of repopulation progressing with time (Zafar et al. 2015; Syedain et al. 2015; Miller et al. 2016; Reimer et al. 2017; Emmert et al. 2018), there are examples, very similar to those mentioned for endothelialization, that show extensive repopulation occurring after relatively short follow-up times (Syedain et al. 2015; Goecke et al. 2018; Fioretta et al. 2020) and limited repopulation in more long-term studies (Theodoridis et al. 2015; Tudorache et al. 2016), independent of the adopted procedure. This indicates that achieving a more complete cellularization is not just a matter of time and suggests that there may be some cellular mechanisms underlying this process that are not yet fully understood and appreciated. Finally, cellular repopulation seems to be less prominent in decellularized xenografts and allografts compared to decellularized TEMs and synthetic grafts (Weber et al. 2013; Theodoridis et al. 2015; Zafar et al. 2015; Syedain et al. 2015; Tudorache et al. 2016; Miller et al. 2016; Reimer et al. 2017; Kluin et al. 2017; Hennessy et al. 2017; Emmert et al. 2018; Goecke et al. 2018; Coyan et al. 2019; Fioretta et al. 2020; van Rijswijk et al. 2020).

The cells found in the interstitium of TEHVs are typically identified as α-SMA-positive cells, such as myofibroblasts and smooth muscle cells (Theodoridis et al. 2015; Tudorache et al. 2016; Reimer et al. 2017; Hennessy et al. 2017; Goecke et al. 2018; Motta et al. 2018; van Rijswijk et al. 2020), although fibroblast cells have also been observed (Theodoridis et al. 2015; Miller et al. 2016). Spatial heterogeneity of these cells in TEHVs is common and does not seem to be correlated with methodology. There is a clear trend of a higher repopulation on the ventricular side of the valve compared to the pulmonary or aortic side (Theodoridis et al. 2015; Tudorache et al. 2016; Reimer et al. 2017; Hennessy et al. 2017; Goecke et al. 2018), as well as a higher cell density in the base and middle of the leaflets compared to the tip (Theodoridis et al. 2015; Syedain et al. 2015; Tudorache et al. 2016; Miller et al. 2016; Goecke et al. 2018; Motta et al. 2018, 2019). This latter finding suggests a dominant role for cellular infiltration from the valve root into the leaflets. However, a few studies using decellularized TEMs have reported a more uniform cellular distribution from leaflet base to tip (Reimer et al. 2017; Emmert et al. 2018). In the study of Reimer et al. (2017) this was achieved after a relatively short follow-up time of 22 weeks, suggesting that infiltrating cells may also originate from the blood. Interestingly, cells were partially absent from the lamina fibrosa of pulmonary valves in some studies (Theodoridis et al. 2015; Tudorache et al. 2016; Goecke et al. 2018). Altogether, these results indicate that the cellular distribution in TEHVs is often dissimilar to native valves and that the underlying mechanisms are still largely unknown.

##### ECM formation

In addition to cellular repopulation, it is important to consider the presence and distribution of matrix components, as these are key contributors to tissue function (Sect. 2.4). Collagen is the most abundant matrix component in all studies that have analyzed the matrix composition (Zafar et al. 2015; Syedain et al. 2015; Tudorache et al. 2016; Reimer et al. 2017; Kluin et al. 2017; Hennessy et al. 2017; Emmert et al. 2018; Goecke et al. 2018; Motta et al. 2018; Lintas et al. 2018; Fioretta et al. 2020; van Rijswijk et al. 2020). However, collagen content has been observed to reach native levels only occasionally (Kluin et al. 2017; Motta et al. 2018) and is often reported as more limited or at lower levels compared to the native situation (Theodoridis et al. 2015; Syedain et al. 2015; Kluin et al. 2017; Goecke et al. 2018; Fioretta et al. 2020), indicating room for improvement. Nevertheless, as synthetic grafts do not possess any initial collagen, the presence of collagen fibers in these grafts at least provides evidence of in situ deposition of collagen (Kluin et al. 2017; Fioretta et al. 2020). This is supported by various studies with decellularized grafts that have found an increase in collagen content in the explanted graft or the presence of procollagen molecules (Syedain et al. 2015; Tudorache et al. 2016; Reimer et al. 2017; Hennessy et al. 2017; Emmert et al. 2018). On the other hand, only a few studies have confirmed in situ deposition of elastin in TEHVs (Reimer et al. 2017; Kluin et al. 2017; Fioretta et al. 2020), although its presence has been shown on many occasions in studies using decellularized scaffolds (Zafar et al. 2015; Syedain et al. 2015; Hennessy et al. 2017; Emmert et al. 2018; Goecke et al. 2018; Motta et al. 2018; Lintas et al. 2018). Even when present, the elastin content is nevertheless only sparse and lower compared to native levels (Reimer et al. 2017; Motta et al. 2018; Lintas et al. 2018; van Rijswijk et al. 2020). Furthermore, in contrast to the spatial distribution of cells in leaflets, clear trends in the distribution of matrix components cannot easily be identified. Only a few studies report more collagen and elastin presence near the base, and decreasing amounts toward the tip (Kluin et al. 2017; Emmert et al. 2018). Also, a higher matrix content is sometimes seen on either the arterial (Kluin et al. 2017) or ventricular (Goecke et al. 2018; Motta et al. 2018) side of the leaflet of a pulmonary valve.

##### Tissue organization

A layered organization of (part of) the leaflets is seen only occasionally (Zafar et al. 2015; Tudorache et al. 2016; Lintas et al. 2018) and is usually absent in in situ TEHVs (Zafar et al. 2015; Reimer et al. 2017; Hennessy et al. 2017; van Rijswijk et al. 2020). In some cases, the presence of a layered structure only represents the maintenance of the original matrix architecture in decellularized xeno- and allografts (Tudorache et al. 2016; Lintas et al. 2018), while in situ layer formation from a synthetic scaffold is reported very rarely (Kluin et al. 2017). Interestingly, in the study of Emmert et al. (2018), one explant displayed a more advanced native-like tri-laminar organization, while other explants lacked such a layered structure and showed, for example, collagen fibers that were less aligned compared to native valves. Other signs of native-like remodeling are occasionally found in TEHVs as well, such as a dense collagen layer on the arterial side (Theodoridis et al. 2015) and elastin presence on the ventricular side of the leaflet (Motta et al. 2018). Nevertheless, a suboptimal organization which does not resemble the native conditions appears to be more common. For example, a number of studies report the presence of collagen or elastin on both sides of the leaflet simultaneously (Theodoridis et al. 2015; Reimer et al. 2017; Kluin et al. 2017; Goecke et al. 2018) or the presence of both collagen and elastin together on the same side of the leaflet (Fig. 1f) (Reimer et al. 2017; Kluin et al. 2017). Both these observations represent an unphysiological organization. Generally, these aspects of unphysiological tissue organization seem to occur independent of the applied procedure and follow-up time (Table 2).

##### Functionality and complications

Overall, in situ heart valve tissue engineering has delivered promising results. An example is the good in vivo performance of engineered pulmonary valve replacements in sheep for up to one year starting from a synthetic scaffold (Kluin et al. 2017) or decellularized TEM (Emmert et al. 2018). However, various complications have been reported as well, which may interfere with optimal and long-term valve functionality. One of the most prominent issues is the progressive development of regurgitation, with moderate to severe cases reported frequently (Miller et al. 2016; Reimer et al. 2017; Kluin et al. 2017; Hennessy et al. 2017; Miyazaki et al. 2017; Soliman et al. 2017; Motta et al. 2018; Lintas et al. 2018; van Rijswijk et al. 2020). Interestingly, there is also quite some variability within experimental groups, with often only one or a few TEHVs developing moderate to severe regurgitation (Zafar et al. 2015; Tudorache et al. 2016; Kluin et al. 2017; Miyazaki et al. 2017; Soliman et al. 2017; Lintas et al. 2018). On a few occasions, the regurgitation worsened over time, potentially due to adverse remodeling (Syedain et al. 2015; Miller et al. 2016; Reimer et al. 2017; Motta et al. 2018). The related issue of leaflet retraction is also still common in recent studies (Tudorache et al. 2016; Reimer et al. 2017; Hennessy et al. 2017) and an important factor compromising valve functionality (Weber et al. 2013; Driessen-Mol et al. 2014; Syedain et al. 2015; Reimer et al. 2017). Calcification is another problem that has been regularly observed in in situ TEHVs (Theodoridis et al. 2015; Zafar et al. 2015; Tudorache et al. 2016; Miller et al. 2016; Reimer et al. 2017; Emmert et al. 2018; Fioretta et al. 2020; van Rijswijk et al. 2020). Other complications such as stenosis (Tudorache et al. 2016; Miller et al. 2016; van Rijswijk et al. 2020) and thrombosis (Tudorache et al. 2016; Fioretta et al. 2020) are much less common and only occur occasionally.

### Challenges in cardiovascular tissue engineering

Despite the promising results obtained via in vitro and in situ approaches, several challenges remain to be overcome. Except for TEBVs exposed to low-pressure conditions (Hibino et al. 2010; Wystrychowski et al. 2014; Lawson et al. 2016), clinical translation of CVTE technologies has been limited. A possible factor that is slowing down the clinical translation is the significant outcome variability of engineered tissues, between and within studies (Visser et al. 2021) (Sect. 3.2.2). For example, both extensive cellular repopulation in short follow-up times and limited repopulation in longer periods have been reported in studies involving in situ TEHVs (Theodoridis et al. 2015; Syedain et al. 2015; Tudorache et al. 2016; Goecke et al. 2018; Fioretta et al. 2020). Similarly, engineered tissues of experimental groups within the same studies often showed different functionality, despite adopting the same procedure in all cases (Zafar et al. 2015; Kluin et al. 2017; Miyazaki et al. 2017; Soliman et al. 2017; Lintas et al. 2018).

Many TEBVs and TEHVs have not only been suboptimal and variable in terms of function, but also with regard to tissue organization. The problems in tissue organization are related to ECM deposition and suboptimal layer formation. In particular, elastin deposition in TEBVs and TEHVs is usually lower compared to their native counterparts (Fig. 1e—f). Specifically, elastin deposition has been almost absent in in vitro TEHVs. A completely native-like organization of ECM components has also not been obtained so far, which may provide an explanation for the observed compromised functionality of TEBVs and TEHVs (Sodian et al. 2000a; Schmidt et al. 2010; Reimer et al. 2017; Kluin et al. 2017). A common issue for both in vitro and in situ TEHVs is also the presence of α-SMA-positive cells, mainly associated with activated VICs (Liu et al. 2007a). These active myofibroblast-like cells continuously proliferate, migrate, remodel ECM, and produce cytokines, which may result in pathological cases such as calcification and leaflet retraction (Jian et al. 2003; Walker et al. 2004; Rutkovskiy et al. 2017). It is not yet clear whether and how the α-SMA-positive cell population can decrease over time to reach homeostasis in engineered valves. Overall, unphysiological cell activities and ECM properties negatively affect the functionality of engineered tissues, which in turn limits the potential for clinical translation.

The outcomes of the studies reviewed in Sects. 3.1 and 3.2 are likely dependent on many factors including the species and implant sites. For example, the animal models used in in situ TEBV studies are mainly mice and rat (Table 1), whereas larger animals like sheep are used in in situ TEHV studies (Table 2). Therefore, the responses might be species-specific. In addition, it is not clear to what extent these animal models are optimal for testing the G&R of TEBV and TEHV and translating the findings into clinical applications.

Another challenge is to show that TEBVs and TEHVs can actually adapt to changing demands, such as in case of somatic growth. Although there are a few studies reporting that TEBVs can grow in diameter (Shinoka et al. 2005; Hoerstrup et al. 2006; Hibino et al. 2010; Syedain et al. 2016), the capacity of TEBVs and TEHVs to grow and adapt is still largely unknown. In addition, the findings in adult models cannot easily be translated in pediatric application because of age-related differences, for example in regenerative capacity and hormone profiles (Ponzi et al. 2020).

To overcome current limitations and improve the organization and function of TEBVs and TEHVs, we need a better and more mechanistic understanding of cardiovascular G&R. A mechanistic understanding and ability to predict G&R can ultimately help us to control the development of engineered tissues and achieve functional tissue organization.

### Computational growth and remodeling models

Given the variability in outcome of experimental studies on CVTE, and the suboptimal tissue organization that is often still obtained (Sects. 3.1, 3.2, and 3.3), there is a clear need for a more detailed understanding of the G&R of engineered cardiovascular tissues to guide future studies. Here, we define *growth* as changes in tissue mass and *remodeling* as changes in tissue structure and/or material properties. Computational models are particularly suited to increase our understanding of tissue G&R, as they are capable of systematically testing hypotheses and thereby providing key insights into some of the mechanisms underlying tissue G&R. In addition, these models may be used to predict tissue G&R, enabling researchers to optimize tissue engineering protocols in a more time- and cost-effective way compared to using empirical trial-and-error approaches alone. In this section, we discuss several computational G&R models and highlight how mechanical stimuli can drive the G&R of blood vessels and heart valves. We also discuss the potential application of G&R models to understand and improve CVTE.

#### Collagen remodeling models

Collagen is the main load-bearing component of cardiovascular tissues. The orientation and degree of anisotropy of collagen fibers are therefore key in determining the tissue mechanical behavior. Consequently, many computational remodeling models have focused mainly on predicting collagen alignment and understanding the mechanisms responsible for collagen remodeling. Early phenomenological models hypothesized that collagen fibers align along or in between the directions of principal stress or strain, which enabled them to successfully predict the collagen organization in heart valves and arteries (Driessen et al. 2003, 2004, 2005, 2008; Boerboom et al. 2003; Baek et al. 2006; Kuhl et al. 2007; Hariton et al. 2007). In more recent studies, models of collagen remodeling have been developed that accounted for the effects of cell behavior, such as contractility and cell alignment, to unravel the underlying biological mechanisms (Loerakker et al. 2014, 2016; Soares et al. 2014; Ristori et al. 2018a). These models suggest that collagen remodeling is driven by mechanical stimuli both directly, via the influence of strain, and indirectly, via mechano-mediated cell behavior. Additionally, it has been predicted that mechanical stimuli provided by hemodynamic loading dominate the cell-mediated collagen remodeling process in TEHVs implanted in the aortic position, whereas the influence of contractility was predicted to be more important for TEHVs implanted in the pulmonary position (Loerakker et al. 2016). To simulate long-term collagen remodeling in heart valves, the influence of topographical stimuli was included in a more recent study as well (Ristori et al. 2018a, 2018b). Simulations with this model revealed that cell traction and reorientation in response to mechanical stimuli can potentially explain the emergence of an anisotropic collagen organization in fetal heart valves, while the coalignment of collagen fibers with cells seems vital for maintaining and reinforcing the adopted collagen organization over time (Ristori et al. 2018a). Taken together, these modeling results suggest a clear and fundamental role for mechano-mediated cellular activity in the process of collagen remodeling in cardiovascular tissues.

#### Tissue growth and remodeling models

In addition to tissue remodeling, many models also incorporate tissue growth. To model the biological growth of a material, two theories are generally adopted, both of which are rooted in continuum mechanics. The first is often referred to as the theory of kinematic growth and was first conceptualized by Skalak et al. (1981; 1982) and later formalized by Rodriguez et al. (1994). According to this theory, growth can be modeled by splitting the deformation of a material into an irreversible growth part, which is typically stress-free, and a reversible elastic part, which does generate stresses. In particular, a multiplicative decomposition of the deformation gradient tensor into a growth tensor and an elastic tensor is usually adopted. The elastic tensor ensures compatibility of the resulting configuration, gives rise to residual stresses, and accounts for elastic deformations resulting from externally applied loads. Whereas this kinematic growth theory generally focuses on the *consequences* of G&R, the theory of constrained mixtures, developed by Humphrey and Rajagopal (2002), places more emphasis on the physiological *process* of G&R. In the latter theory, biological tissues are modeled as a mixture of different constituents (e.g., collagen fibers, elastin, cells) and G&R is simulated by accounting for the production and removal of individual constituents. These constituents may each possess distinct mechanical properties, production and removal rates, and evolving stress-free configurations. Nevertheless, once they are deposited into the tissue, they are assumed to deform together with the tissue as a whole. The theory of constrained mixtures is especially relevant for providing an increased understanding into the G&R of engineered tissues as underlying mechanisms can be accommodated more easily when experimental data are available (Gleason and Humphrey 2005; Humphrey et al. 2007). However, this approach is computationally much more expensive compared to the kinematic growth theory.

#### Effects of mechanical stimuli

By implementing these theories, computational models have confirmed that mechanical stimuli play a fundamental role in tissue G&R and helped uncover several underlying mechanisms. For example, for arteries, simulations have shown that stress correlates better with growth than strain (Taber and Humphrey 2001). This suggests that arterial growth may be stress-regulated, a hypothesis that has been adopted by many subsequent theoretical studies (Humphrey and Rajagopal 2003; Gleason and Humphrey 2005; Baek et al. 2006, 2007; Kuhl et al. 2007; Valentín et al. 2009, 2011; Valentín and Humphrey 2009). Furthermore, both intramural stresses and wall shear stresses can contribute to tissue G&R in these models. The latter can drive tissue remodeling via vasoactive molecules that regulate matrix turnover (Valentín et al. 2009). In addition, shear stresses sensed by VSMCs arising from interstitial flow have also been shown to affect G&R (reviewed in Shi and Tarbell 2011) and have been modeled in a number of studies (Tada and Tarbell 2000, 2002). More specifically, it is likely not stress itself but rather deviations in stress from a homeostatic target value that drive G&R of arterial tissue, as shown by multiple studies simulating the adaptation of arteries in response to altered pressure and/or flow (e.g., due to hypertension) (Humphrey and Rajagopal 2003; Gleason and Humphrey 2005; Valentín et al. 2009; Valentín and Humphrey 2009; Karšaj et al. 2010). Nevertheless, it still remains uncertain whether stress or some other quantity acts as a target variable for mechanical homeostasis, thereby driving tissue G&R (Eichinger et al. 2021). Similar mechanisms have successfully been applied to model arterial adaptation after balloon angioplasty or stenting (Kuhl et al. 2007), as well as the progression of arterial diseases such as cerebral aneurysm (Baek et al. 2006; Cyron et al. 2014) and vasospasm (Baek et al. 2007; Humphrey et al. 2007). The model of Rachev et al. (2011) revealed more details by considering radial variations in arterial G&R and showed that *local* deviations in stresses can control remodeling and induce a heterogeneous distribution of collagen and elastin in the arterial wall. In addition to stress, material stiffness is also an important determinant of G&R and simulations have suggested that anisotropic stiffness induces anisotropic growth in arteries, resulting in a preference of the material to grow in the direction of the lowest stiffness (Braeu et al. 2019).

#### Applications in cardiovascular tissue engineering

To illustrate the benefits of adopting G&R models in tissue engineering applications, we discuss a few inspiring examples from the literature. G&R models have, for instance, been employed to perform parametric studies with the aim of optimizing the properties of synthetic scaffolds for tissue-engineered vascular grafts (Courtney et al. 2006a; Engelmayr and Sacks 2006a; Miller et al. 2015; Szafron et al. 2017a, 2019). This enabled the prediction of several sets of parameter values that result in favorable conditions, such as minimal compliance mismatch between the scaffold and host tissue (Miller et al. 2015; Szafron et al. 2019), as well as some specific techniques that may be employed to prevent high stiffness deviations during graft development which are detrimental to tissue outcome (Miller et al. 2015). In addition, it was shown that cells infiltrating the core of a bi-layered vascular graft were often stress-shielded, which could lead to a decreased matrix production and compromised mechanical integrity of the neovessel (Szafron et al. 2017b). Other models have helped researchers to design scaffolds for heart valve tissue engineering with a favorable degree of fiber anisotropy resulting in similar mechanical properties to native tissues (Courtney et al. 2006b; Engelmayr and Sacks 2006b). This was achieved by numerically predicting the mechanical properties of these scaffolds based on fiber orientation. More key aspects of neotissue development were captured by the model of Best et al. (2019), which suggested that improving the properties of newly formed collagen reduces the likelihood of dilation and graft rupture, while the stiffness and degradation profile of scaffolds have little effect. Furthermore, Szafron and Khosravi et al. (2018) simulated both mechano-driven and immuno-driven growth of neovessels, suggesting that mechano-driven growth alone is not enough to explain the experimentally observed development of vascular grafts, and that a delayed and moderate immune response is desirable. Finally, computational modeling was able to successfully predict the performance and remodeling of TEHVs and motivated the design of scaffolds which resulted in favorable valves with significantly improved long-term in vivo functionality (Loerakker et al. 2013; Emmert et al. 2018).

The examples in this section demonstrate the ability of computational G&R models to increase our understanding of G&R of vessels and valves and to guide and optimize tissue engineering protocols. These models have consistently identified mechanical stimuli as one of the major driving factors for the G&R of cardiovascular tissues, and a clear trend toward more mechanistic modeling is observed. It is important to note that many of the models discussed in this section have been validated qualitatively by comparing model outcomes to experimental or clinical observations. Nevertheless, more thorough quantitative validation is needed in many cases to ensure the accuracy and robustness of these models. Most of these models have not yet focused on integrating cell–cell signaling, despite this being an important mechanism for cell behavior and tissue G&R. The inclusion of cell–cell signaling may open new possibilities to guide and optimize the organization of engineered tissues. Therefore, further efforts are needed to unravel the underlying mechano-mediated biological mechanisms, such as cell behavior and cell–cell signaling, and incorporate these into computational frameworks.

## The effects of mechanical cues on cell behavior

Mechanical cues have important roles in the regulation of cellular processes that are responsible for vascular and valvular G&R (Taber 2001; Humphrey 2006; Humphrey et al. 2014). Cardiovascular cells respond to both dynamic mechanical cues arising from the hemodynamic loading conditions acting on cardiovascular tissues and static mechanical cues such as the stiffness of the microenvironment (reviewed in Chaudhuri et al. 2020; Wang et al. 2020). Due to the dynamic nature of the cardiovascular system, in this section, we discuss the experimental findings that show how dynamic mechanical cues, particularly stress and strain, affect the behavior of vascular endothelial and smooth muscle cells, as well as valvular endothelial and interstitial cells. Particular attention is given to the effects of stress and strain on cellular processes that are important for G&R, such as cell orientation, ECM synthesis and organization, proliferation, and apoptosis. Cell migration is another important cellular process that is essential for obtaining proper recellularization of the implanted grafts. Cell migration is mechano-regulated as well, as extensively reviewed elsewhere (Li et al. 2005; Chi et al. 2014; Campinho et al. 2020).

### Vascular endothelial cells

ECs lining the luminal surface of blood vessels are exposed to shear stress due to blood flow and circumferential cyclic strain resulting from the pulsatile blood pressure. The blood flow is usually unidirectional and laminar, with a mean wall shear stress of 1–2 Pa (10–20 dynes/cm^2^) in the straight sections of arteries and 0.1–0.6 Pa (1–6 dynes/cm^2^) in veins (Roux et al. 2020). Yet, spatial variations in wall shear stress are also observed, especially in regions with curvatures and bifurcations. Cells in large arteries also experience cyclic circumferential stress of around 100–150 kPa at mean arterial pressure, resulting in 10–15% strain (van Haaften et al. 2017). These strain levels are not the same as what the cells feel locally. This is the reason why in in vitro studies, 5–10% strain is considered physiological, while 20% strain and higher magnitudes are considered pathological (Charbonier et al. 2019). Both shear stress and cyclic strain are important direct regulators of EC morphology and physiological function. Prolonged changes in these mechanical cues alter EC morphology and function, which in turn causes vascular abnormalities such as intimal hyperplasia and atherosclerosis (GimbroneJr and García-Cardeña 2016).

ECs primarily sense the shear stress through a thin glycocalyx layer that coats their luminal membrane. Glycocalyx also acts as a mechanotransducer of shear stress to the endothelial cytoskeleton and initiator of the biochemical responses (Tarbell et al. 2005; Weinbaum et al. 2007). Animal studies have indicated that vascular ECs orient in the direction of blood flow, and that this orientation is not uniform in arterial branches where the flow is turbulent (Langille and Adamson 1981; Nerem et al. 1981). These observations have been corroborated by in vitro experiments providing more controlled flow environments. Specifically, compared to static conditions, ECs exposed to steady laminar flow in vitro have been shown to elongate, obtain an ellipsoid shape (Dewey et al. 1981), and align in the direction of flow (Dewey 1984; Levesque and Nerem 1985). Moreover, it has been demonstrated that ECs reorganize the actin and myosin stress fibers (Dewey 1984) as well as the underlying ECM (Wechezak et al. 1985; Thoumine et al. 1995a) in the direction of flow. Pulsatile laminar flow appears to have the same effect on EC alignment and actin fiber organization compared to steady laminar flow conditions (Helmlinger et al. 1991). The morphological changes and the reorganization of the cytoskeleton and ECM depend on the applied levels of shear stress and the time of exposure (Franke et al. 1984). Increasing the levels of shear stress and exposure time upregulates actin fiber formation and organization, resulting in better aligned ECs in the direction of flow. On the other hand, such an orientation and ECM reorganization are not observed under oscillatory shear stress (Helmlinger et al. 1991; Thoumine et al. 1995b) and multidirectional shear stress (Mohamied et al. 2015; Ghim et al. 2018). Interestingly, ECs exposed to oscillatory shear stress and multidirectional shear stress are randomly oriented and demonstrate a similar cobblestone morphology as in static conditions.

Shear stress also influences the proliferation and apoptosis of vascular ECs. Steady laminar shear stress with higher magnitudes than physiological levels and pulsatile shear stress inhibit DNA synthesis and EC proliferation by inhibiting the transition from the G0/G1 toward the S phase in the cell cycle (Levesque et al. 1990; Akimoto et al. 2000). Low levels of steady laminar flow, on the other hand, lead to similar EC proliferation compared to no flow conditions. (Dewey et al. 1981; Akimoto et al. 2000). Laminar shear stress also suppresses apoptosis (Kaiser et al. 1997, 1999, Dimmeler et al. 1996). On the contrary, the absence of laminar shear stress triggers EC apoptosis in organ cultures and in freshly isolated ECs cultured in vitro (Kaiser et al. 1997, 1999), thereby showing the importance of laminar shear stress for EC survival. In contrast to the results obtained with laminar flow, turbulent flow appears to increase EC proliferation and apoptosis (Dardik et al. 2005). This proliferation effect is independent from applied shear stress levels and exposure time (Davies et al. 1986). Overall, these results show that physiological levels of laminar flow inhibit EC proliferation and apoptosis, promoting homeostasis in the vessel wall, while sub-physiological levels of laminar shear stress and turbulent shear stress promote EC proliferation and apoptosis. This could be correlated with plaque formation and atherosclerosis development (Zarins et al. 1983; Cunningham and Gotlieb 2005).

Cyclic strain is another mechanical cue affecting vascular EC proliferation and apoptosis. However, current literature studies are inconsistent with respect to the specific effect of strain on EC proliferation. This inconsistency might be caused by differences in cell sources, cyclic strain levels, and exposure time between studies. For example, Sumpio et al. (Sumpio et al. 1987) reported that bovine aortic ECs increased DNA synthesis and proliferation when exposed to 10% cyclic strain at 0.05 Hz after 1 day compared to static conditions, while Woodell et al. (Woodell et al. 2003) reported that application of 4% cyclic strain at 0.1 Hz for 4 h decreased DNA synthesis in bovine aortic ECs. In addition, rabbit aortic ECs have been shown to decrease cell proliferation and DNA synthesis when exposed to 18% cyclic strain, but demonstrated an increase in cell proliferation at 24% and 27% cyclic strain compared to unloaded samples (Upchurch et al. 1997), suggesting that the effect of cyclic strain on EC proliferation is not necessarily monotonic. Further systematic studies are therefore necessary to determine and understand how strain regulates EC proliferation. With regard to apoptosis, experiments have indicated that 6% and 10% cyclic strain protects ECs from apoptosis induced by TNF-α and serum depletion (Haga et al. 2003; Liu et al. 2003). However, 20% cyclic strain appeared not to have the same effect (Liu et al. 2003). These findings suggest that physiological levels of cyclic strain protect arterial ECs from apoptosis, while pathological levels promote cell apoptosis.

Regarding cell morphology, ECs elongate and orient perpendicular to the direction of stretch (Wang et al. 2001; Moretti et al. 2004), which is accompanied by the alignment of microtubules (Ives et al. 1986). In addition, ECs form actin stress fibers in response to cyclic strain (Sumpio et al. 1988a) and (re)orient these filaments and their cell shape perpendicular to the stretch direction (Dartsch and Betz 1989, Yoshigi et al. 2003), which is known as a strain avoidance response (Buck 1980; De and Safran 2008; Hsu et al. 2009). This (re)orientation response to cyclic stretch is consistent with the vessel structure and the (re)orientation response to shear stress. Strain in the vessel wall is circumferential; hence, the tendency of ECs to (re)orient in directions perpendicular to the applied cyclic strain leads to EC alignment in the axial direction of the vascular wall, which agrees with the preferred direction of ECs due the presence of shear stress. Therefore, both shear stress and strain seem to act together in aligning ECs in the direction of flow.

The possible synergistic effects of both shear stress and strain on the orientation of ECs are supported by in vitro studies. When pulsatile shear stress and uniaxial cyclic strain are applied together to ECs, the elongation and alignment of ECs in the direction of shear stress and perpendicular to cyclic strain is enhanced (Zhao et al. 1995). In particular, actin stress fibers become thicker and more aligned when compared to the individual application of each stimulus. In another study, fluid shear stress alone had the largest effect on cell elongation followed by the shear stress and biaxial cyclic strain together (Meza et al. 2016). However, the differences in outcome of these studies might be related to the levels of shear stress and the direction of cyclic strain (uniaxial or biaxial), since it is known that equibiaxial strain does not cause an alignment (Meza et al. 2016; Sinha et al. 2016). In addition, when the cells are exposed to anisotropic biaxial strain and shear stress simultaneously, the alignment response of ECs is dominated by anisotropic strain when the shear stress levels are lower than physiological levels, whereas physiological levels of shear stress dominate the alignment response (Sinha et al. 2016). Therefore, it seems that different levels of shear stress determine the strain-mediated EC alignment.

### Vascular smooth muscle cells

VSMCs can express different phenotypes related to vascular G&R. VSMCs generally display a quiescent contractile phenotype in healthy adult vessels. Upon changes in hemodynamic conditions and biochemical stimuli, VSMCs can switch to a synthetic phenotype that is characterized by increased proliferation and ECM deposition (Owens et al. 2004). VSMCs are located in the tunica media and are predominantly exposed to cyclic circumferential stress and strain resulting from the pulsatile blood flow (Anwar et al. 2012). This mechanical stress is thought to regulate vascular G&R by affecting VSMC orientation, proliferation, apoptosis, and phenotype, as well as VSMC-mediated ECM synthesis and degradation.

Many in vitro two-dimensional cell culture studies have shown that VSMCs demonstrate a strain avoidance response by aligning perpendicular to the direction of uniaxial cyclic stretch (Sumpio and Banes 1988; Kanda et al. 1992; Standley et al. 2002; Chen et al. 2003; Li et al. 2003). This alignment response is faster and stronger for higher strain magnitudes and frequencies (Kanda et al. 1992; Liu et al. 2008), corresponding to physiological conditions. Moreover, VSMCs reorient toward a random organization again if they are subjected to static conditions after 48 h of stretch, showing that the alignment response is reversible (Standley et al. 2002). Cell orientation is also influenced by structural cues such as collagen and scaffold fibers (Guido and Tranquillo 1993; Fioretta et al. 2014), patterned substrates (Ray et al. 2017; Buskermolen et al. 2020), and grooved geometries (Lamers et al., 2010). This phenomenon is known as contact guidance (Dunn and Heath 1976). Its effects on cellular orientation are extensively reviewed elsewhere (Tamiello et al. 2016; Leclech and Villard 2020).

Cyclic strain also affects ECM synthesis. In fact, it has been shown that strain increases the production of collagen and fibronectin in VSMCs (Leung et al. 1976; Sumpio et al. 1988b; O’Callaghan and Williams 2000; Stanley et al. 2000). This synthesis dependence seems to require long-term exposure to strain, as it takes 4 to 5 days to become apparent (Sumpio 1988c, O’Callaghan 2000). Higher magnitudes of strain increase this response (O’Callaghan 2000), while variations in strain frequency do not affect the results (Leung et al. 1976; Sumpio et al. 1988b). Cyclic strain also increases matrix metalloproteinase (MMP) activity, particularly MMP-2 (O’Callaghan and Williams 2000; Grote et al. 2003; Seo et al. 2013). MMP-2 is known to enable vascular remodeling by degrading the existing ECM, while the new ECM is being synthesized and organized (Galis and Khatri 2002). The increase in MMP-2 production has also been validated in vivo with a rat model of aortic banding (Liu et al. 2015). The pressure overload via banding upregulated MMP-2 activity as well as the production of collagen I and III. Overall, these results suggest that cyclic strain promotes ECM synthesis and degradation in VSMCs.

The observed effects of cyclic strain on VSMC apoptosis have been rather consistent. Specifically, it has been shown that this mechanical stimulus increases apoptosis in VSMCs independent of strain magnitude, direction, and cell source (Sotoudeh et al. 2002; Wernig et al. 2003; Morrow et al. 2005; Su et al. 2006; Guha et al. 2011; Cheng et al. 2012; Song et al. 2012a). On the other hand, VSMCs have shown a heterogeneous response to strain in terms of proliferation. Determining the causes of such variability is challenging. For example, cyclic strain increases proliferation in rabbit aortic and bovine aortic SMCs compared to static conditions (Birukov et al. 1995; Li et al. 2003; Chahine et al. 2012), while it decreases the proliferation of porcine aortic SMCs (Sumpio and Banes 1988). These findings suggest that the response of VSMCs might be species-specific; however, this hypothesis is not consistent with the variation that can for example be seen by investigating the cyclic strain responses of rat and human VSMCs. In human aortic SMCs, it has been reported that cyclic strain can either increase (Song et al. 2012a) or have no effect on proliferation (O’Callaghan and Williams 2000) compared to static cases. Similarly, for rat VSMCs, some studies reported that cyclic strain increases proliferation compared to unloaded samples (Wilson et al. 1993; Standley et al. 1999; Song et al. 2012b), while others reported the opposite (Chapman et al. 2000; Morrow et al. 2005; Guha et al. 2011) at similar magnitudes of strain but different time points. These disagreements do not appear to depend on the application of equibiaxial versus uniaxial strain (Standley et al. 1999; Chapman et al. 2000; Morrow et al. 2005; Song et al. 2012a). Therefore, further studies are necessary to identify the parameters affecting the proliferation response of VSMCs. For example, future studies might investigate if these variations are caused by differences in terms of blood vessel location in the arterial tree or different media supplements.

The phenotype of VSMCs is also affected by cyclic strain. Specific phenotypic markers such as α-SMA, calponin, smooth muscle protein 22-alpha, and smooth muscle myosin heavy chain have been used to determine the contractile phenotype of VSMCs (Owens et al. 2004). Cyclic strain has been reported to both upregulate (Reusch et al. 1996; Tock et al. 2003; Yao et al. 2014) and downregulate (Butcher et al. 2006; Hu et al. 2014; Rodríguez et al. 2015; Wan et al. 2015) the expression of contractile proteins compared to static controls. An upregulation in contractile marker expression could be associated with lower strain magnitudes (Tock et al. 2003) and higher frequencies applied (Yao et al. 2014) with regard to the studies showing a downregulation in contractile marker expression (Butcher et al. 2006; Hu et al. 2014; Rodríguez et al. 2015). The differences in applied strain directions, exposure time, cell types, and surface coatings should also be taken into account. In addition, the mechanisms involved in the phenotypic switch of VSMCs via mechanical stimulation should be further investigated.

### Valvular endothelial cells

VECs have different orientation responses compared to vascular ECs. VECs cover the surface of valve leaflets and are exposed to shear stress and strain in circumferential and radial directions. Despite this duality in terms of mechanical stimuli, studies have mainly focused on the effect of shear stress on VEC behavior. In vivo observations have shown that VECs align circumferentially on both sides of aortic valve leaflets, hence perpendicular to flow (Deck 1986). In agreement with that, in vitro studies have shown that VECs align perpendicular to unidirectional laminar flow (Mahler 2014) and parallel to uniaxial strain (Balachandran et al. 2011), differently than vascular ECs (Butcher et al. 2004). This differential alignment response is thought to depend on different spatial arrangements of focal adhesion proteins and different signaling pathways activated in VECs versus vascular ECs.

Increasing evidence also suggests that there are differences in VECs in terms of morphology, gene and protein expression, and mechanical properties based on their location ( aortic versus ventricular side of the valve leaflet) (Holliday et al. 2011; Bischoff and Aikawa 2011; Miragoli et al. 2014; Mongkoldhumrongkul et al. 2018). VECs on the ventricular side of the leaflets are more elongated and flattened compared to the cuboidal shape of VECs on the aortic side (Maron and Hutchins 1974; Bischoff and Aikawa 2011). Moreover, VECs on the ventricular surface are relatively stiffer than the VECs on the aortic surface, which might be related to the presence of high and unidirectional shear stress on the ventricular side and low oscillatory shear stress on the aortic side of the valve in vivo (Miragoli et al. 2014). In addition, an increase in collagen and GAG production on the aortic side caused by oscillatory flow cannot be replicated by applying an identical flow pattern to the ventricular side of the valve (Mongkoldhumrongkul et al. 2018). Thus, VECs located on different sides of the valves respond differently to shear stress. The mechanosensitive mechanisms regulating these different responses should be further investigated.

### Valvular interstitial cells

VICs are located throughout the three layers of the valve leaflets. VICs exhibit a high degree of phenotypic plasticity. Quiescent VICs are generally found in healthy adult tissues and are characterized by low proliferation levels, ECM synthesis, and remodeling. These cells can switch phenotype toward activated VICs, which are characterized by increased proliferation, as well as ECM production and remodeling (Rabkin-Aikawa et al. 2004). A maladaptive activation of VICs can result in an osteoblastic differentiation, which is mainly associated with valve dysfunction (Liu et al. 2007a). This phenotypic activation is characterized by increased α-SMA (Liu et al. 2007a; Latif et al. 2015). Cyclic strain regulates the phenotypic activation of VICs, in such a way that increasing the strain magnitude increases α-SMA expression, thus VIC activation (Ferdous et al. 2013; Ayoub et al. 2017). The activation of VICs with pathological strain levels (15%) is also related to osteogenic gene expression and calcification (Balachandran et al. 2010; Ferdous et al. 2013). Physiological strain levels (10%), on the contrary, maintain tissue homeostasis (Ferdous et al. 2013; Ayoub et al. 2017; Bogdanova et al. 2018).

VICs are highly sensitive to the magnitude, direction, and the duration of strain. For example, cell proliferation and apoptosis increase in VICs with the magnitude of cyclic stretch they are exposed to (Balachandran et al. 2009, 2012). The nuclear aspect ratio of mitral VICs, which is a metric of cell deformation, increases with increasing strain (Ayoub et al. 2017, 2021). In addition, increasing anisotropy of biaxial cyclic strain also upregulates proliferation and apoptosis (Gould et al. 2012). In terms of ECM synthesis and remodeling, VICs respond to cyclic strain by increasing total collagen synthesis, depending on the magnitude and duration of stretch (Balachandran et al. 2006, 2009, 2012; Ku et al. 2006; Ayoub et al. 2017). In particular, when physiological (10%) and pathological strain levels (20% and 30%) are compared, it can be seen that physiological strain levels maintain the remodeling activity with respect to the homeostatic conditions, while pathological strain levels increase collagen production and MMP expression (Balachandran et al. 2009; Ayoub et al. 2017). VICs exposed to cyclic anisotropic biaxial strain generally align perpendicular to the first principal strain direction, which also aligns the collagen fibers (Gould et al. 2012). On the other hand, GAG synthesis has been reported to decrease (Gupta et al. 2009) or not change (Ayoub et al. 2017) with applying 10% strain compared to static conditions, but increases at pathological strain levels (30%) (Ayoub et al. 2017). Elastin levels do not change upon applying 10% cyclic stretch (Balachandran et al. 2006; Ayoub et al. 2017) and decrease with 30% strain (Ayoub et al. 2017).

**Fig. 2:**
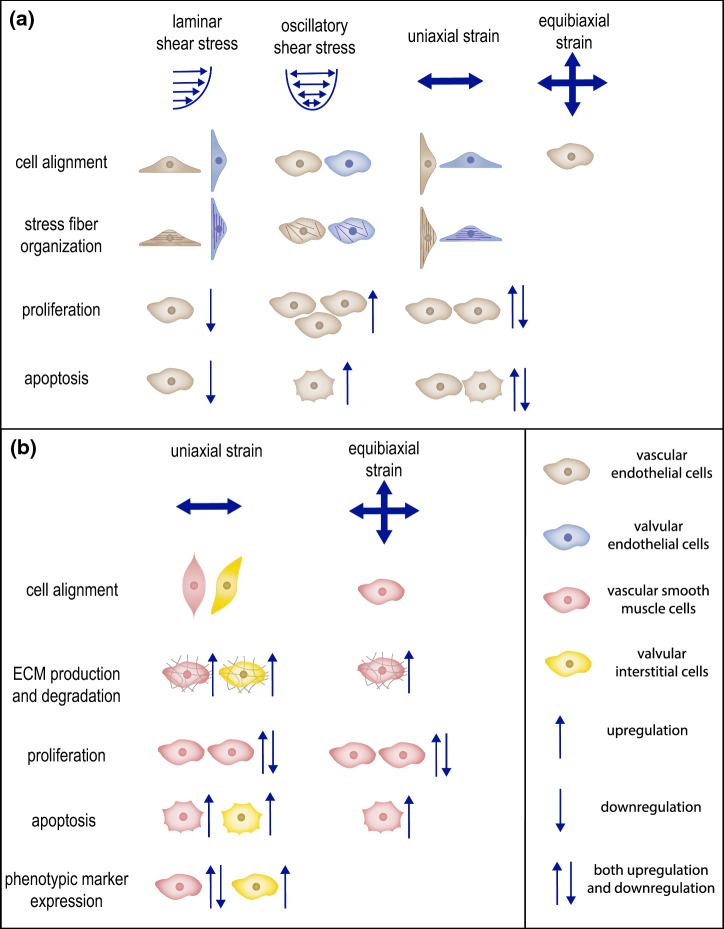
The effects of different mechanical cues on vascular and valvular cell behavior **a** the effects of laminar and oscillatory shear stress as well as uniaxial and equibiaxial strain on vascular EC and VEC behavior, **b** the effects of uniaxial and equibiaxial strain on VSMC and VIC behavior

In summary, mechanical cues that are applied on vascular and valvular cells differently affect cellular processes responsible for cardiovascular G&R (Fig. 2). Understanding these relationships is important for regulating remodeling and organization in the context of CVTE. It is clear that vascular ECs align parallel to flow and VSMCs align perpendicular to the direction of stretch, which is consistent with the vessel wall structure and the applied hemodynamic loads. VECs show a different orientation response than vascular ECs by aligning perpendicular to flow. The alignment of the cells in response to mechanical cues is in accordance with the alignment of their microtubules and stress fibers. Laminar shear stress is needed to maintain vascular homeostasis, while oscillatory shear stress increases EC proliferation and apoptosis, thereby potentially promoting G&R (Humphrey 2006). With regard to VSMCs, even though strain is known to increase VSMC apoptosis, understanding how VSMC proliferation and phenotypic switches are regulated by strain requires further investigation as previous experimental observations have been inconsistent. Increased mechanistic understanding of how vascular and valvular cells regulate cardiovascular G&R and incorporating these insights into scaffold designs will allow more control over tissue formation and therefore better TEBV and TEHV functionality.

## Mechano-regulated cell–cell signaling pathways

Apart from the influence of mechanical cues on tissue G&R (Sect. 3.4) and cell behavior (chapter 4), cell behavior is also strongly influenced by cell–cell signaling. An increasing number of these signaling pathways have been identified as mechanosensitive. Cell–cell signaling might therefore be a key underlying mechanism regulating the G&R of tissues in response to mechanical stimuli. This is relevant in the context of tissue engineering, as an increased understanding of these mechanisms could enable us to identify ways of controlling tissue G&R and cell behavior and thereby improve tissue engineering outcomes. In this section, we review mechanosensitive juxtacrine signaling pathways, in which direct cell–cell contact is required, and mechanosensitive paracrine signaling pathways, occurring at larger length scales. In particular, we focus our attention on signaling pathways that are known to be both mechanosensitive and important in the development of cardiovascular tissues.

### Notch signaling

**Fig. 3:**
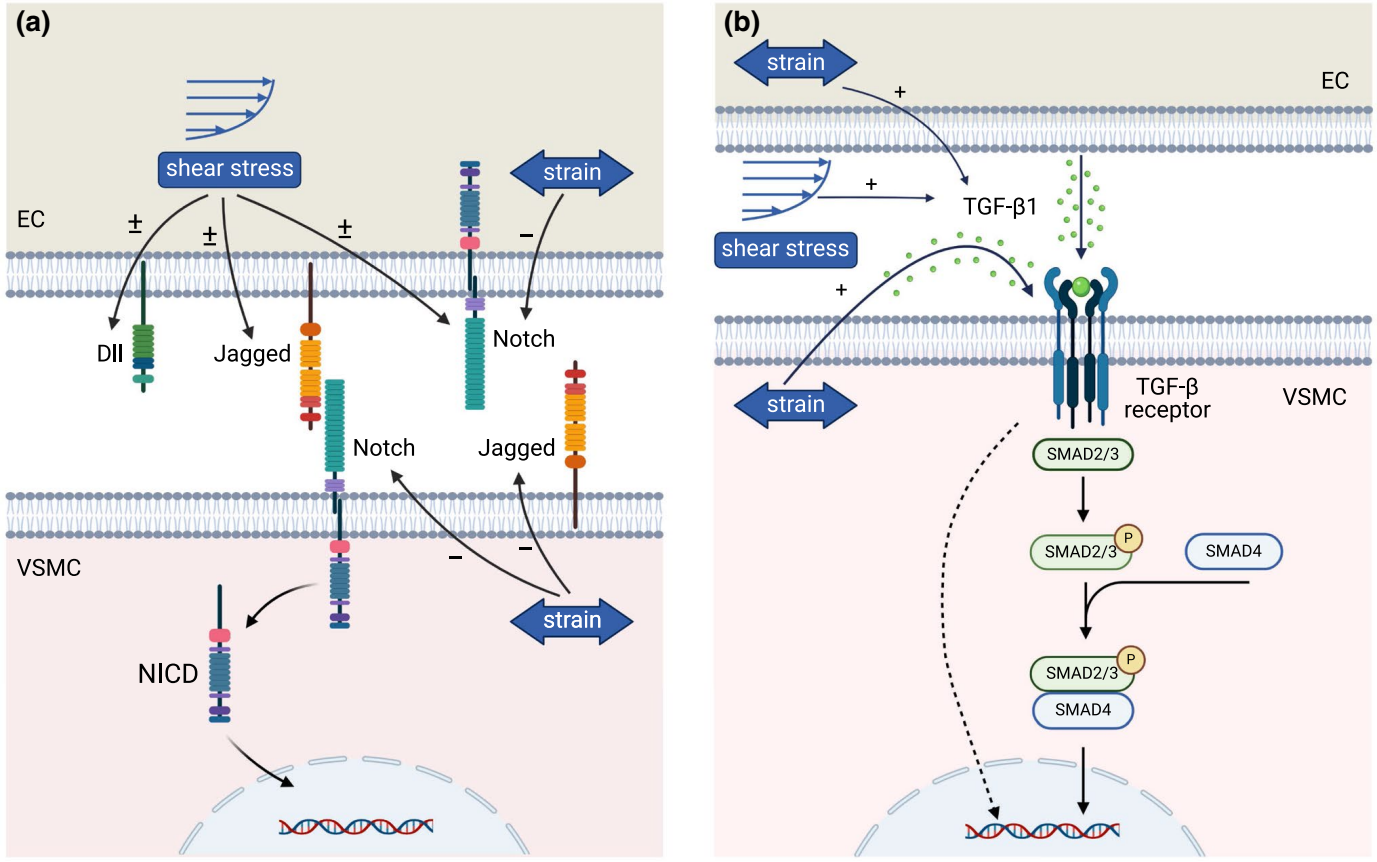
The effects of mechanical stimuli on cell–cell signaling pathways in ECs and VSMCs **a** the Notch signaling pathway where a Jagged ligand from the EC binds to a Notch receptor on the VSMC, resulting in the translocation of NICD to the nucleus. In the EC strain downregulates Notch expression, while shear stress can either up- or downregulate the expression of Notch, Dll, and Jagged. In the VSMC, strain downregulates the expression of both Notch and Jagged. **b)** the TGF-β signaling pathway where the TGF-β ligand binds to the TGF-β receptor and activates canonical SMAD or noncanonical (dashed arrow) cascades. Shear stress and strain upregulate TGF-β1 release from ECs, and strain upregulates TGF-β1 release from VSMCs to create an autocrine feedback (created with BioRender.com)

Notch is an evolutionarily conserved signaling pathway involved in cell fate determination in most tissues of the human body. In mammals, the pathway consists of four receptors (Notch1 to Notch4), two Jagged ligands (Jagged1 and Jagged2), and three Delta-like ligands (Dll1, Dll3, and Dll4). All receptors and ligands are presented on the cell membranes, requiring direct cell–cell contact for signaling to occur. Notch is activated when a ligand of one cell binds to a receptor of a neighboring cell, which results in the proteolytic cleavage of the Notch intracellular domain (NICD) in the receiving cell (Fig. 3a). NICD then translocates to the nucleus where it acts as a cofactor for transcription of Notch target genes. Receptors can bind not only to ligands from a neighboring cell (trans-interactions) but also to ligands from the same cell (cis-interactions). This latter phenomenon is generally thought to have an inhibitory effect (Sprinzak et al. 2010), although recent reports affirm that it can also lead to Notch activation (Nandagopal et al. 2019).

Notch signaling plays an essential role in the development of almost all tissues of the human body (Artavanis-Tsakonas et al. 1995; Gridley 2007; Rostama et al. 2014). It is a main regulator of cell fate decisions, proliferation, apoptosis, boundary formation, and regeneration (Gridley 2007; de la Pompa 2009; MacGrogan et al. 2018). It is therefore not surprising that the Notch pathway is also crucially involved in both the development and homeostasis of the cardiovascular system (Iso et al. 2003; Gridley 2007; de la Pompa 2009; Baeten and Lilly 2017; MacGrogan et al. 2018). In the vasculature, Notch signaling regulates angiogenesis, EC migration and proliferation, barrier function, arterial-venous specification of both ECs and VSMCs, and modulation of the VSMC phenotype (Iso et al. 2003; Gridley 2007; Phng and Gerhardt 2009; Baeten and Lilly 2017; Polacheck et al. 2017; Mack et al. 2017). During cardiac development, the Notch pathway controls the proliferation and differentiation of cardiomyocytes, epithelial–mesenchymal transition, trabeculation, and the formation and morphogenesis of cardiac valves (de la Pompa 2009; MacGrogan et al. 2018).

One of the primary features of the Notch pathway is its ability to establish patterns in cell populations by regulating cell differentiation (Collier et al. 1996; Hamada et al. 2014; Shaya et al. 2017; Corson et al. 2017). Via Notch signaling, cells can instruct their immediate neighbors to adopt either a different or a similar phenotype. These processes are called lateral inhibition and lateral induction, respectively, and rely on negative and positive feedback loops. In the case of lateral inhibition, Notch activation results in the downregulation of ligand expression in signal receiving cells, preventing them from becoming signal sending cells themselves, thus creating patterns of alternating signal sending and receiving cells in the cell population (Sjöqvist and Andersson 2019). During lateral induction, on the other hand, Notch activation promotes ligand expression in receiving cells, enabling them to send signals to their neighbors, resulting in a cascade of Notch activation in which all cells adopt a similar phenotype (Sjöqvist and Andersson 2019). These processes may be one of the reasons for Notch ubiquity in organisms, as they enable the transmission of local stimuli to larger length scales in a versatile and controlled fashion.

Increasing evidence suggests that the Notch pathway is influenced by a wide variety of external bio-chemo-mechanical stimuli. Examples include interactions with ECM components, crosstalk with other signaling pathways, mechanical cues, and pathological cues such as hypoxia and hyperglycemia (reviewed in LaFoya et al. 2016). At the same time, Notch activity impacts these stimuli by regulating cell behavior. Therefore, the Notch pathway may serve not only as a facilitator of direct cell–cell communication, but also as an integrator of micro-environmental cues (LaFoya et al. 2016). This makes the Notch pathway an interesting factor to consider in the context of tissue engineering, as it potentially allows us to steer signaling interactions by manipulating these stimuli and thereby control tissue G&R. In the following paragraphs, we describe the effects of mechanical stimuli on Notch signaling. For a more detailed description of the molecular mechanisms underlying Notch mechanotransduction, we refer the reader to one of our recent reviews (Stassen et al. 2020).

#### Effects of mechanical stimuli on Notch signaling in ECs

Shear stress on vascular ECs resulting from blood flow is an important regulator of Notch signaling (Fig. 3a). An increase in Notch activation is generally seen in response to shear stress in human ECs from different locations in the vascular tree (Polacheck et al. 2017; Fang et al. 2017; Mack et al. 2017) as well as in bovine (Wang et al. 2007) and murine (Tu et al. 2014) ECs. Results are less conclusive, however, when the receptor and ligand specific responses to shear stress are investigated. Upregulation of Notch1 (Tu et al. 2014; Jahnsen et al. 2015; Mack et al. 2017) and Dll4 (Tu et al. 2014; Jahnsen et al. 2015; Polacheck et al. 2017; Fang et al. 2017; Driessen et al. 2018) in shear stressed ECs are common. Other Notch-related proteins that are known to respond to shear stress are Notch2 (Wang et al. 2007), Notch4 (Tu et al. 2014; Jahnsen et al. 2015; Fang et al. 2017), Dll1 (Wang et al. 2007; Tu et al. 2014; Jahnsen et al. 2015; Fang et al. 2017), Jagged1 (Fang et al. 2017; Driessen et al. 2018), and Jagged2 (Fang et al. 2017). These are typically upregulated upon exposure to shear stress, although in some cases also downregulated. For a more detailed overview of the effects of mechanical stimuli on Notch signaling, see Table 3. These differential outcomes may be explained by variations in cell type, shear stress magnitude, and exposure time. Indeed, the shear stress response of Notch signaling is known to depend on both the magnitude (Jahnsen et al. 2015; Fang et al. 2017) and exposure time (Mack et al. 2017; Driessen et al. 2018) of shear stress. An interesting example is the study by Fang et al. (2017) who showed that Notch activation in human umbilical vein ECs increased with increasing shear stress up to a critical value, after which it decreased with increasing shear stress, resulting in an inverse V-shaped profile. Interestingly, this critical value of shear stress at which Notch activation is maximal, corresponds well to the physiological value in arterial ECs. This suggests an important role for shear stress-induced Notch signaling in arterial remodeling, as the activation of Notch near this critical shear stress value promoted EC cycle arrest and subsequent arterial specification (Fang et al. 2017).

**Table 3:** Overview of the effects of mechanical stimuli on the Notch signaling pathway in various cell types

Cell type	Loading conditions	Results	Substrate	References
Rat EC	Shear stress from in vivo blood flow (increased from 0.4 to 4.6 Pa over 84 days)	Upregulation of Notch1 (after 3 days) and Notch4 (after 21 days) Upregulation of Dll1 and Dll4 Increasing shear stress increases number of interactions between Notch1 and Dll1, Dll4 and Jagged1 and between Notch4 and Dll1, Dll4 and Jagged1	Glass slides coated with 1% gelatin	Tu et al. (2014)
Bovine Aortic EC	Constant shear stress (1.5 Pa)	Activation of Notch signaling (no direct measurement of Notch receptors or ligands)	In vivo experiments	Wang et al. (2007)
Human Aortic EC	Laminar shear stress	Downregulation of Notch1 (10 min – 4 h) Upregulation of Notch1 (12 h – 48 h) Upregulation of Notch1 target genes HES1, NRARP, and FABP4 (after 12 h) Increase in NICD levels (after 24 h)	6-well Bioflex plates (Flexcell International Corp) coated with fibronectin	Mack et al. (2017)
Human Abdominal aortic EC	Laminar shear stress (0.1—1.5 Pa)	Upregulation of Notch1 (0.1 – 0.5 Pa) No change in Notch1 (> 1 Pa)	Culture slides coated with 4% rat tail collagen type I	Jahnsen et al. (2015)
	Shear stress from fully oscillatory flow (0 ± 0.3 Pa)	Upregulation of Notch1		
	Shear stress oscillatory flow with some retrograde flow (0.2 ± 0.3 Pa)	Upregulation of Notch1		
	Shear stress from fully pulsatile flow (shear stress 0.5 ± 0.3 Pa)	No change in Notch1		
Human Umbilical Vein EC	Laminar shear stress (0.3—5 Pa, for 24 h)	Increase in NICD content (after 1, 3, and 6 h) Increase in NICD content depended on shear stress magnitude (maximum at physiological value of 1.8 Pa)	Ibidi μ-Slides and 6-well plates coated with gelatin	Fang et al. (2017)
Human Umbilical Vein EC	Cyclic strain (10%, 1 Hz, for 24 h)	Initial upregulation of Notch1, Notch4, and target gene Hrt (maximum after 4 h), followed by return to control levels (after 24 h) Initial increase in Notch1 ICD content (after 4 h) and Notch4 ICD content (after 2 h), followed by decrease in Notch1 ICD and Notch 4 ICD content (both after 24 h)	Plastic slides pre-treated with fibronectin	Morrow et al. (2007)
Rat VSMC	Uniform equibiaxial cyclic strain (0—15%, 1 Hz, for 24 h)	Downregulation of Notch1, Notch3, Jagged1, and target genes Hrt1, Hrt2, Hrt3, HES1, and HES5 Downregulation of Notch3 and HES5 is time- and force-dependent	Microfluidic PDMS device bonded to glass, injected with a collagen type I solution	Morrow et al. (2005)
Human Coronary Artery VSMC	Radial cyclic strain (1—9%, 1 Hz, for 24 h)	Strain-dependent downregulation of Notch3, Jag1, and target genes HES1, HEY1, and HEY2 No changes in Notch1, Notch2, and Dll1	Ibidi slides coated with collagen type IV	Loerakker et al. (2018)

Shear stress is not the only mechanical stimulus experienced by vascular ECs, as they are also subjected to cyclic stress and strain (Sect. 2.4). The effect of cyclic strain on the Notch pathway in ECs has been studied by Morrow et al. (2007), who cultured human umbilical vein ECs exposed to a cyclic strain of up to 10% for 24 h. They reported a time-dependent response, characterized by an initial upregulation in the levels of Notch1 and Notch4 mRNA and intracellular domain, followed by a return to baseline values after 24 h. This temporal increase in Notch activation appeared to be sufficient to enhance the angiogenic activity in ECs.

#### Effects of mechanical stimuli on Notch signaling in VSMCs

While most of the research on unraveling the interplay between Notch signaling and mechanical stimuli has been focused on ECs, some efforts have also been made to reveal the effects of mechanical cues on Notch signaling in VSMCs. The results show a remarkable contrast with ECs, as mechanical stimuli seem to cause a decrease in the expression of Notch pathway proteins in VSMCs (Fig. 3a). Morrow et al. (2005) reported a reduction in the mRNA expression of Notch1, Notch3, Jagged1, and downstream targets in VSMCs subjected to cyclic strain. The downregulation of Notch1 and target gene *Hes5* was shown to depend on both the amplitude of cyclic strain and the duration of exposure. Interestingly, this downregulation was accompanied by inhibited proliferation and enhanced apoptosis of VSMCs, suggesting a regulatory role for Notch signaling in strain-induced VSMC behavior. Similar results were obtained more recently by Loerakker et al. (2018), who found a downregulation in gene expression of Notch3, Jagged1, and downstream targets proportional to the magnitude of the applied cyclic strain. On the other hand, no effects were observed in the expression of Notch1, Notch2, and Dll1. See Table 3 for a more detailed overview.

### Eph-ephrin signaling

Eph receptors are part of a large family of receptor tyrosine kinases and transduce signals by binding to ephrin ligands. There are 14 Eph receptors and 8 ephrin ligands (Flanagan and Vanderhaeghen 1998), classified into subclasses A and B (Gale et al. 1996). In general, EphA and EphB receptors interact with ephrin-A and ephrin-B ligands, respectively (Gale et al. 1996), although there are some exceptions (Gale et al. 1996; Himanen et al. 2004). Similar to the Notch pathway, cell–cell contact between adjacent cells is generally required to activate the Eph-ephrin pathway (Davis et al. 1994), as both ligand and receptor are membrane-bound. Longer-distance signaling has also been observed through cell protrusions (Cayuso et al. 2016) or exosomes (Gong et al. 2016). Eph-ephrin binding can result in signal transduction into the receptor-expressing cell (forward signaling), into the ligand-expressing cell (reverse signaling) or into both cells (bi-directional signaling) (Kania and Klein 2016; Niethamer and Bush 2019). This range of signaling modes makes the Eph-ephrin pathway very versatile, and it is involved in various processes in almost all tissues and organs (Kania and Klein 2016; Niethamer and Bush 2019). Its main roles are associated with cell migration (Krull et al. 1997; Arthur et al. 2011), and tissue segregation and boundary formation (Xu et al. 1999; Cooke et al. 2005; Rohani et al. 2011, 2014). In the cardiovascular system, Eph-ephrin signaling is key for the establishment of boundaries between arterial and venous cells, termed arterial-venous specification (Tallquist et al. 1999; Adams 2003; Aitsebaomo et al. 2008; Michaelis 2014; Kania and Klein 2016). Additionally, the Eph-ephrin pathway has been connected to angiogenesis (Tallquist et al. 1999; Michaelis 2014), vascular morphogenesis (Adams et al. 2001), the regulation of vessel tone (Wu et al. 2012), and the migration, adhesion, and proliferation of ECs (Michaelis 2014).

The expression of Eph receptors and ephrin ligands in the vasculature is influenced by mechanical cues, and this mechanosensitivity mainly impacts arterial-venous specification. For example, Xue et al. (2017) found that culturing murine endothelial progenitor cells on stiff substrates enhanced the expression of ephrin-B2, associated with an arterial fate, and attenuated the expression of EphB4, associated with a venous fate, compared to soft substrates. This indicates that the stiffness of the micro-environment might modulate arterial-venous specification via Eph-ephrin signaling. They identified the Ras/Mek pathway as the main mechanotransduction mechanism and regulator of ephrin-B2 and EphB4. In addition to stiffness, shear stress has also been revealed as a mechanoregulator of Eph and ephrin expression. To study the remodeling of a vein graft used as an arterial bypass, human saphenous veins were exposed to arterial levels of shear stress ex vivo, resulting in a decrease in EphB4 expression (Berard et al. 2013; Model et al. 2014). Interestingly, the expression of ephrin-B2 showed no significant difference upon application of arterial shear stress alone (Berard et al. 2013; Model et al. 2014) and only decreased when the pressure was also increased to arterial levels (Berard et al. 2013). These results indicate that arterial shear stress alone causes a loss of venous identity in these cells, without inducing a gain in arterial identity. It is important to note that, despite the clear effects of shear stress on Eph-ephrin regulation, it is not clear whether EphB4 and ephrin-B2 are direct mechanosensors to shear stress, or whether they are downstream components of a larger mechanotransduction cascade (Model et al. 2014), which would be similar to the stiffness-dependent regulation of ephrin-B2 and EphB4 governed by Ras/Mek signaling (Xue et al. 2017).

### TGF-β superfamily signaling

In addition to juxtacrine signaling, cells can communicate over longer distances via paracrine signaling. Cells can produce a signal via the secretion of paracrine factors that can diffuse over relatively short distances to induce changes in nearby cells. Paracrine factors bind to their corresponding receptors of the signal receiving cell and initiate a series of reactions called signal transduction cascades within the receiving cell, changing its behavior (Gilbert 2010). Paracrine signaling pathways are important regulators of cardiovascular development and homeostasis. A well-established member of paracrine signaling, transforming growth factor-β (TGF-β) superfamily signaling, is known to be mechanosensitive in vessels and valves (Li et al. 2019; Souilhol et al. 2020; Hiepen et al. 2020). In addition, it is involved in the regulation of cell behavior via crosstalking with other signaling pathways (Tang et al. 2010; Martin-Garrido et al. 2013; Chen et al. 2016). In this section, we discuss the mechanosensitive regulation of TGF-β superfamily signaling and its implications for vascular and valvular G&R.

In mammals there are at least thirty ligands of the TGF-β superfamily, including three TGF-β ligands (TGF-β1, TGF-β2, TGF-β3) and bone morphogenic proteins (BMPs) (Schmierer and Hill 2007). The transmembrane receptors of the TGF-β superfamily are categorized as type I and type II receptors based on their structural differences. In the canonical pathway, each ligand of the TGF-β superfamily binds to a specific combination of type I and type II receptors to initiate signaling and activate the SMAD family of transcription factors (Heldin et al. 1997). Once the ligands bind to the receptors, type II receptors phosphorylate the intracellular domain of type I receptors, which in turn phosphorylates receptor-regulated SMAD (R-SMAD) proteins. Phosphorylated R-SMAD proteins form a complex with SMAD4, and this complex translocates into the nucleus where it acts as a transcription factor to regulate the expression of target genes (Fig. 3b).

Both TGF-β and BMP ligands activate similar mechanisms; however, the receptors that they bind to, and the associated phosphorylated R-SMAD proteins, are different. Type I and type II receptors of TGF-β ligands are TβR-I/ALK5 and TβR-II, whereas those of BMP ligands are BMPR-IA/ALK3, BMPR-IB/ALK6, ActR-I/ALK2; BMPR-II, ActR-II, ActR-IIB (Shi and Massagué 2003). In addition, the binding of TGF-β ligands to specific receptors causes the phosphorylation of SMAD2 and SMAD3, whereas SMAD1 and SMAD5 are phosphorylated when BMP ligands bind. Nevertheless, despite these differences, both SMAD2/3 and SMAD1/5 generate a complex with SMAD4 (Heldin et al. 1997).

TGF-β superfamily signaling is a highly conserved pathway that controls a diverse set of cellular processes, such as cell growth, differentiation, cell fate determination, matrix production, and apoptosis. It has important roles in pattern formation during development, tissue remodeling, and homeostasis (Heldin et al. 1997; Shi and Massagué 2003). In the cardiovascular system, TGF-β superfamily signaling is, for example, necessary for endothelial–mesenchymal transformation for cardiac cushion formation, angiogenesis, VSMC recruitment to the vessels, EC and VSMC proliferation and migration, VSMC differentiation, vascular stabilization, and cardiovascular homeostasis (Dickson et al. 1995; Oshima et al. 1996; Bonyadi et al. 1997; Galvin et al. 2000; Nakajima et al. 2000; Carvalho et al. 2007; Ramsauer and D’Amore 2007; Chen et al. 2009). Dysregulation of TGF-β superfamily signaling is associated with the development of several cardiovascular anomalies, including atherosclerosis, aneurysms, cardiac fibrosis, and calcification of the valves (Jian et al. 2003; Loeys et al. 2006; Gomez et al. 2009; Van De Laar et al. 2011).

TGF-β and BMP signaling are influenced by mechanical cues (Fig. 3b). Laminar shear stress significantly increases TGF-β1 and TGF-β3 gene and protein expression in vascular ECs compared to static conditions (Ohno et al. 1995; Cucina et al. 1998; Song et al. 2000; Negishi et al. 2001; Walshe et al. 2013). In addition, ECs release TGF-β1 and TGF-β3 upon the application of shear stress. The effect of the released TGF-β1 on VSMC behavior is not clear. For instance, it has been shown that TGF-β1 inhibits VSMC growth and migration by downregulating DNA synthesis and causing a cell-cycle arrest (Owens et al. 1988; Morisaki et al. 1991; Halloran et al. 1995; Cucina et al. 1998; Seay et al. 2005). On the other hand, an effect of TGF-β1 on VSMC proliferation has also been reported (Stouffer and Owens 1994; Schulick et al. 1998; Suwanabol et al. 2012; Calvier et al. 2017). In addition, the TGF-β1 released by ECs under low-shear (pathological) stress conditions does not participate in the paracrine control of VSMCs (Qi et al. 2011). Both SMAD-dependent and SMAD-independent TGF-β signaling pathways are responsible for regulating the VSMC behavior, and the exact mechanisms for controlling the cell behavior are still unclear.

Oscillatory shear stress upregulates BMP4, leading to proliferation and the secretion of inflammatory adhesion molecules in vascular ECs (Sorescu et al. 2003; Zhou et al. 2013). In agreement with this, arterial ECs increase BMP4 expression at sites of disturbed flow compared to sites of laminar flow (Chang et al. 2007). Other findings suggest a link between shear stress-dependent BMP activation and disease development. For example, BMP2 and BMP4 are expressed in ECs of atherosclerotic plaques (Dhore et al. 2001), while they are absent in healthy segments of human arteries (Zhou et al. 2012). VSMCs exposed to cyclic strain increase TGF-β1 mRNA and protein expression compared to static controls (O’Callaghan and Williams 2000; Mata-Greenwood et al. 2003, 2005), which in turn upregulates ECM production in VSMCs via an autocrine mechanism (O’Callaghan and Williams 2000). Cyclic strain also increases the production of TGF-β by vascular ECs (Baker et al. 2008; Dong et al. 2019). Interestingly, strain-induced endothelial TGF-β signaling controls VSMC proliferation via an autocrine feedback mechanism (Baker et al. 2008). In particular, autocrine TGF-β signaling in ECs regulates perlecan secretion in response to strain, which in turn inhibits the proliferation of VSMCs.

In valvular tissues, low and oscillatory shear stress upregulates TGF-β1, BMP4, and inflammatory gene expression in VECs (Sucosky et al. 2009; Mahler et al. 2014). These results are in accordance with the higher levels of SMAD1 and SMAD5, which are activated by BMP signaling, in human calcified aortic valves compared to healthy valves (Ankeny et al. 2011). In addition, cyclic strain increases BMP2, BMP4, and TGF-β1 expression in aortic VICs in a stretch magnitude-dependent manner, while the lowest expression has been detected at physiological strain levels (Ferdous et al. 2013). Pathological levels of strain are also associated with increased cellular apoptosis in VICs and valve calcification via BMP signaling (Balachandran et al. 2010). Overall, mechanical stimuli are important mediators of paracrine TGF-β and BMP signaling in cardiovascular homeostasis and disease. Shear stress and strain upregulate TGF-β in vascular ECs to regulate VSMC growth, and low and oscillatory shear stress upregulate BMP expression in ECs and VECs, related to the occurrence of pathologies. Strain-dependent changes in TGF-β and BMP signaling also alter VSMC and VIC behavior. Thus, the interplay between mechanical cues and TGF-β superfamily signaling is an important regulator of vascular and valvular cell behavior, which could be a target for improving and controlling engineered cardiovascular tissues.

### Computational cell–cell signaling models

Computational models that describe juxtacrine and paracrine signaling generally aim at gaining a more complete and detailed understanding of the mechanisms of various signaling pathways. In this section, we discuss models for Notch signaling, Eph-ephrin signaling, and TGF-*β* signaling, with a special emphasis on models that incorporate the impact of these signaling pathways on tissue G&R.

#### Notch signaling models

Numerous computational models for Notch signaling have been developed to understand and predict Notch-regulated cell fate decisions and distributions (Binshtok and Sprinzak 2018). They generally employ a set of ordinary differential equations that describes the time evolution of Notch pathway components, such as receptors and ligands, by accounting for their production, degradation, and interactions. Given the key role of Notch in tissue patterning, most Notch signaling models have focused on understanding the signaling dynamics underlying different patterns. Model complexity has increased over the years by considering an increasing number of biological phenomena.

Early models have shown that fine-grained patterns of cells with alternating phenotypes, also known as “salt-and-pepper” patterns, can be obtained from both Notch lateral inhibition and cis-inhibition (Collier et al. 1996; Sprinzak et al. 2010, 2011). Recall from Sect. 5.1 that Notch lateral inhibition is a mechanism in which cells instruct their immediate neighbors to adopt a different phenotype, which requires regulation of Notch components at the gene transcriptional level, resulting from Notch trans-activation (Collier et al. 1996). Cis-inhibition, on the other hand, simply arises from the mutual inhibition of ligands and receptors within the same cell (Sprinzak et al. 2010). When coupled with trans-interactions, cis-inhibition enriches and accelerates pattern formation (Sprinzak et al. 2010, 2011; Formosa-Jordan and Ibañes 2014) and facilitates sharp tissue boundary formation (Sprinzak et al. 2011). More intricate cell patterns, such as cell clusters, stripes, and labyrinths, can be modeled by considering long-range cell–cell signaling occurring through filopodia (Chen et al. 2014; Vasilopoulos and Painter 2016; Hadjivasiliou et al. 2016). Other models have considered the influence of other cell properties such as cell division and migration (Hunter et al. 2016; Tóth et al. 2017) or cell geometry and contact area (Khait et al. 2016; Akanuma et al. 2016; Shaya et al. 2017; Guisoni et al. 2017) on Notch signaling. These studies revealed, for example, that Notch signaling can regulate the timing of cell differentiation and division to create more ordered patterning (Hunter et al. 2016) and that contact area and cell size can bias cell fate decisions (Shaya et al. 2017). In addition to patterning of static cells, lateral inhibition models can be adopted to study dynamic processes, such as angiogenesis (Bentley et al. 2008, 2009, 2014; Jakobsson et al. 2010; Vega et al. 2020) and the development of multicellular structures (Mulberry and Edelstein-Keshet 2020). Whereas these lateral inhibition models mostly focus on interactions between Notch and Delta, a number of models have also included other ligands, such as Jagged (Petrovic et al. 2014; LeBon et al. 2014; Boareto et al. 2015a, 2015b). This inclusion of Jagged enables the simulation of lateral induction, in which Notch signaling induces neighboring cells to adopt similar fates (Sect. 5.1).

Some Notch models are now considering mechanical stimuli as an influential feature of the cellular environment (Riahi et al. 2015; Loerakker et al. 2018; van Engeland et al. 2019; Ristori et al. 2020). Building on the model of Boareto et al. (2015b), for example, a recent model was used to investigate Notch signaling in the arterial wall by simulating Notch interactions between ECs and VSMCs (Loerakker et al. 2018). The experimentally derived influence of strain on the synthesis of Notch components in VSMCs (Sect. 5.1), and the correlation between Notch activation and VSMC phenotypes, was incorporated into the Notch model. Importantly, with these additions, the model could predict the homeostatic thickness of several types of native human arteries. This suggests that Notch mechanosensitivity may be a key regulator in the establishment and maintenance of arterial homeostasis and highlights the important role of the interplay between mechanical stimuli and cell–cell signaling in tissue G&R. These examples illustrate that computational Notch models enable the prediction and understanding of some crucial aspects of tissue G&R, such as cell patterning, division, and phenotype, thereby emphasizing the potential of adopting such models in future tissue engineering studies to improve tissue organization.

#### Eph-ephrin signaling models

Given the crucial role of Eph-ephrin signaling in boundary formation (Sect. 5.2), it is not surprising that computational models for Eph-ephrin signaling have mainly focused on this phenomenon (Wong et al. 2010; Aharon et al. 2014; Taylor et al. 2017). These models typically adopt agent-based formulations and simulate cell segregation, clustering, and patterning by accounting for differences in adhesive and repulsive properties between cell populations, which are assumed to be regulated by Eph-ephrin signaling (Wong et al. 2010; Aharon et al. 2014; Taylor et al. 2017).

For example, Wong et al. (2010) showed that differential adhesion between cell populations in the intestinal crypt is crucial for sharp boundary formation and the positioning and migration of cells. Eph-ephrin signaling in the model regulates cell adhesion properties phenomenologically, with interactions between Eph and ephrin decreasing the adhesion strength of cells. A similar approach was adopted by Aharon et al. (2014), who simulated a net force of attraction and repulsion between cells, where attraction is attenuated when Eph-ephrin interactions take place. The model predicts segregation of initially intermingled cell populations resulting from differences in adhesive properties, in good agreement with experimental results (Aharon et al. 2014).

Together, these models show that the complexity of Eph-ephrin signaling can be captured well by computational models, enabling accurate simulation of cell segregation and boundary formation. Nevertheless, computational models of Eph-ephrin still lack the consideration of mechanics as an influential factor on signaling dynamics. For example, changes in Eph and ephrin content have been shown to be regulated by mechanical stimuli (Sect. 5.2). As cell adhesive properties are directly linked to Eph and ephrin content (Wong et al. 2010) and signal strength (Aharon et al. 2014) in the respective models, this suggests that mechanical cues may have an important effect on cell adhesion and consequently on cell segregation and migration, which is worth investigating in future studies.

#### TGF-β signaling models

Computational models of TGF-β signaling can be developed following different strategies. Some models operate on a single cell level and are concerned mainly with the molecular dynamics of the pathway itself (Zi et al. 2012; Nicklas and Saiz 2013; Vizan et al. 2013). A second type of model focuses on tissue-level consequences of TGF-β signaling, which is more relevant in the context of tissue G&R and will therefore be the main topic of this subsection. These models assume that TGF-β regulates the production of tissue components, such as collagen (Aparício et al. 2016; Marino et al. 2017; Keshavarzian et al. 2018, 2019; Khosravi et al. 2020; Irons and Humphrey 2020; Irons et al. 2021). Two main approaches are typically adopted by these models: i) a rule-based or logic-based approach (Keshavarzian et al. 2018, 2019; Irons and Humphrey 2020; Irons et al. 2021) or ii) a system of differential equations to describe the kinetics of signaling molecules (Aparício et al. 2016; Marino et al. 2017; Khosravi et al. 2020). In these models, TGF-β signaling is often part of a more extensive network of signaling pathways and molecules, such as matrix metalloproteinase, platelet derived growth factors, and interleukins (Aparício et al. 2016; Marino et al. 2017; Keshavarzian et al. 2018, 2019; Khosravi et al. 2020; Irons and Humphrey 2020; Irons et al. 2021). Similar to the computational G&R models discussed in Sect. 3.4, these models are typically validated only qualitatively by comparing the simulation results to experimental findings and quantitative validation is a crucial next step in future studies (Aparício et al. 2016; Keshavarzian et al. 2018, 2019; Irons and Humphrey 2020; Irons et al. 2021). Finally, it is important to note that these models typically assume that the characteristic timescale of cell–cell signaling is much smaller than that of tissue G&R (Marino et al. 2017; Irons and Humphrey 2020; Irons et al. 2021).

Using such as modeling approach, Marino et al. (2017) simulated pathophysiological arterial remodeling as a result of increased macrophage activity. This suggested a protective role of TGF-β signaling as it reduced MMP-driven matrix degradation and promoted VSMC-driven matrix deposition to approximately re-establish homeostatic conditions (Marino et al. 2017). Hence, the model was able to capture the role of paracrine signaling in arterial remodeling and describe its consequences for tissue structure and mechanics. However, this model did not account for the direct influence of tissue mechanics on the cell–cell signaling pathways. Mechanical stimuli were included in two recent TGF-β signaling models of G&R (Khosravi et al. 2020; Irons and Humphrey 2020), extending the modeling capabilities and increasing the range of scenarios that can be simulated. Importantly, Khosravi et al. (2020) simulated the in vivo development of a neovessel from a polymeric scaffold by describing tissue production as a function of scaffold design and pharmacological interventions. The simulation results suggest that treatment with TGF-β inhibitors can improve the vessel’s patency and reduce the risk of compliance mismatch by suppressing the immune response and reducing the production of stiff collagen (Khosravi et al. 2020).

While these models (Khosravi et al. 2020; Irons and Humphrey 2020) account for the effects of mechanical stimuli on cell–cell signaling, these stimuli are fully prescribed. The models do not include feedback in the other direction, from the *altered* mechanical state of the tissue to cell–cell signaling. Such a feedback mechanism may be critical for capturing long-term tissue G&R and the effects of sustained chemical or mechanical perturbations. This was demonstrated by the study of Irons et al. (2021) in which their previous signaling model (Irons and Humphrey 2020) was coupled to a constrained mixture model for G&R (see Sect. 3.4). This allows continuous feedback between tissue-level mechanics and cell-level signaling activity and enables the simulation of tissue remodeling in response to sustained changes in blood pressure or flow (Irons and Humphrey 2020). Other models have also included feedback between tissue mechanics and cell–cell signaling (Aparício et al. 2016; Keshavarzian et al. 2018, 2019), which similarly enabled them to investigate long-term G&R, in these cases in the context of aneurysm development (Aparício et al. 2016) and in vitro tissue engineering (Keshavarzian et al. 2019). These models also elucidate the role of TGF-β in these processes. In particular, TGF-β signaling was shown to stabilize aneurysm development by inducing fibroblasts to increase collagen production (Aparício et al. 2016) and a decrease in TGF-β signaling was demonstrated to significantly slow down the growth of a tissue-engineered vascular graft (Keshavarzian et al. 2019).

Together, these models have increased our understanding of the role of TGF-β signaling in G&R of cardiovascular tissues by enabling us to study TGF-β signaling in scenarios that have not been investigated experimentally and predict resulting cell and tissue behavior in previously unexplored conditions. They have also demonstrated the value of adopting computational approaches in CVTE, for example by simulating the role of cell–cell signaling in neotissue development to identify beneficial pharmacological interventions (Khosravi et al. 2020). Furthermore, they have shown that incorporating the effects of mechanical stimuli on cell–cell signaling, such as TGF-β, can provide a key mechanistic explanation of mechano-regulated tissue G&R, especially when bi-directional feedback between tissue mechanics and cell–cell signaling is included.

In conclusion, the studies discussed in this section demonstrate that computational signaling models can increase our understanding of cell–cell signaling in the context of various aspects of tissue G&R, such as cell patterning, differentiation, proliferation, migration, and matrix production. The incorporation of mechanical stimuli in some recent Notch models and various TGF-β models has emphasized the importance of mechanics in tissue G&R and increased our understanding of underlying cell–cell signaling mechanisms and how we can manipulate these to achieve more organized and functional engineered tissues. In addition to the models focusing on blood vessels discussed here, interest in modeling the complex mechano-regulated signaling network of VICs is also growing (Howsmon and Sacks 2021). We therefore propose that more signaling models should include the effects of mechanical cues and that cell–cell signaling should be considered more in mechanical G&R models. This would begin to satisfy the need of a more mechanistic description of G&R, observed in Sect. 3.4. In addition, attention should be given to including bi-directional feedback mechanisms between mechanics at the tissue scale and signaling activity at the cell scale, which are currently absent from most models. This feedback could enable long-term processes to be simulated, as demonstrated by several models (Aparício et al. 2016; Keshavarzian et al. 2019; Irons et al. 2021). Together, these two additions would provide a more detailed description of signaling dynamics and increase the range of behavior that can be modeled. This is valuable for CVTE as it may enable the identification of techniques to control tissue G&R and the resulting outcome of engineered tissues.

## Summary and future perspectives

Cardiovascular tissue engineering aims to regenerate functional blood vessels and heart valves, either in vitro or in situ, to treat various cardiovascular diseases. Despite promising results, this approach has not yet found wide-spread clinical application. The main limitations preventing this include the considerable variability between study outcomes, with some cases exhibiting suboptimal functionality, especially long-term, leading to various complications. In addition, the adaptive capabilities of TEBVs and TEHVs have not been clearly demonstrated yet. Our incomplete understanding of the processes and mechanisms underlying the G&R of engineered cardiovascular tissues hinders the discovery of solutions to overcome these limitations. The wide array of available materials, approaches, and techniques further complicates the search for optimal outcomes. Importantly, while native blood vessels and heart valves display a highly organized and layered structure, crucial to accommodate the hemodynamic loads and ensure functionality, this level of organization is often not seen in engineered blood vessels and heart valves. The importance of tissue organization for native tissues, together with the suboptimal organization of engineered cardiovascular tissues, leads to the hypothesis that the current limitations of CVTE may be overcome by achieving a more native-like organization. To this aim, the G&R processes of cardiovascular engineered tissues need to be better understood and guided.

The main mediator of tissue G&R is cell behavior, which refers to processes such as proliferation, apoptosis, migration, differentiation, and ECM synthesis. It is well accepted that cell behavior is highly influenced by mechanical stimuli in cardiovascular tissues (chapter 4). However, the underlying biological mechanisms of this mechano-regulation are still largely unclear. Cell–cell signaling pathways have also been shown to be sensitive to mechanical stimuli (Sects. 5.1, 5.2 and 5.3), which makes them a promising candidate to form the link between mechanical stimuli and cell behavior, and consequently provide a biological explanation for mechano-mediated G&R of cardiovascular tissues.

Collectively, the studies that we have discussed in chapters 4 and 5 reveal that cell–cell signaling and cell behavior are highly context-specific and often remarkably sensitive to changes in the type, magnitude, and duration of mechanical stimuli. This shows that cell behavior is very versatile and highly tuned to the cells’ local environment and mechanical stimuli to which they are exposed. It also shows that cells are part of sophisticated regulatory systems, with sensitive mechanical feedback loops, which play a vital role in tissue G&R and, particularly, in establishing and maintaining tissue homeostasis. To investigate and understand this complex network with variable cell responses in future tissue engineering studies and computational models, a systems biology approach and context-specific experimental data are required. In addition, models should be sufficiently flexible to describe G&R for a wide range of environmental conditions.

**Fig. 4:**
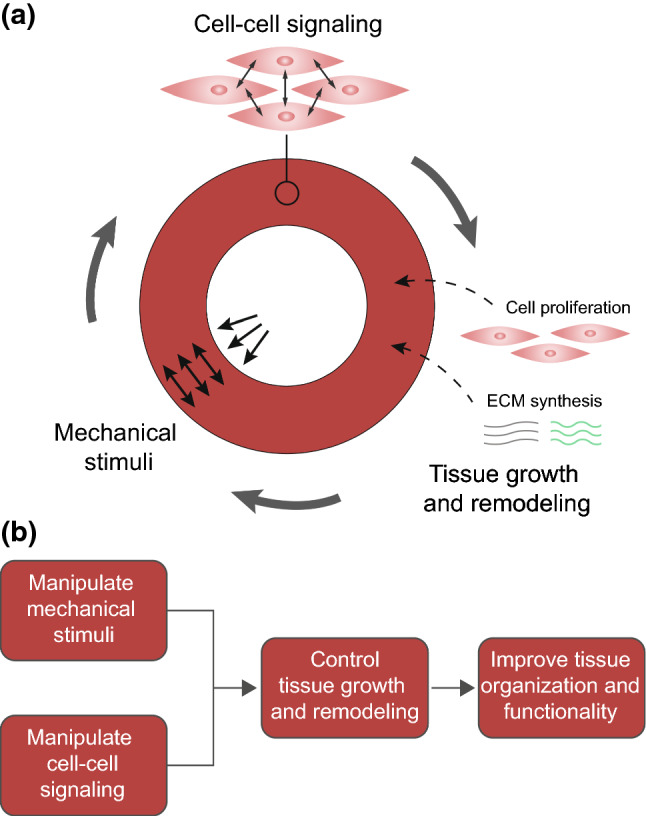
**a** Visualization of the interplay and feedback between mechanical stimuli, cell–cell signaling, and tissue G&R in a blood vessel. **b** This interplay forms the basis of a proposed strategy to improve the outcome of future CVTE studies

As reviewed in chapter 3, the limitations of current tissue-engineered constructs can probably be overcome, or at least reduced, by improving tissue G&R to establish a more native-like tissue organization. Given the strong effects of mechanical stimuli on cell–cell signaling pathways (Sects. 5.1, 5.2, and 5.3) and cellular behavior (chapter 4), we propose that mechano-regulation of cell–cell signaling is an important factor to consider in future tissue engineering studies. In particular, by investigating mechano-regulated cell–cell signaling, a more detailed understanding of the mechanisms underlying mechano-mediated G&R of blood vessels and heart valves can be obtained. This knowledge can reveal novel methods to control tissue G&R via direct or indirect interventions in the signaling pathways (Fig. 4b). Such an increased level of control over tissue G&R in concert with the development of predictive computational models of cell-mediated G&R would enable the identification of promising changes to tissue engineering protocols that can improve tissue organization and associated function, and subsequently accelerate clinical translation.

Deliberate manipulations of signaling pathways have already been recognized as an attractive strategy for tissue engineering (Carlson 2007; Zohorsky and Mequanint 2020). For example, scaffolds could be biologically activated with signaling molecules or growth factors to artificially induce or inhibit cell–cell signaling interactions in infiltrating cells and thereby control cell behavior to mediate tissue G&R. It has been shown that various signaling molecules, such as TGF-β and Notch pathway ligands, can be immobilized onto biomaterial surfaces (Mann et al. 2001; Carlson 2007; Putti et al. 2019a, 2019b; Zohorsky and Mequanint 2020). This technique has already been adopted by several studies to promote the differentiation of mesenchymal stem cells (Wen et al. 2014; Dishowitz et al. 2014) and epithelial stem cells (Beckstead et al. 2006) using immobilized Jagged ligands. This Jagged-induced differentiation was critical for continued tissue development, thereby demonstrating the potential of signaling manipulations for future tissue engineering approaches.

Alternatively, the mechanosensitivity of cell–cell signaling pathways could be utilized to indirectly control signaling activity, and consequent tissue G&R, by influencing the mechanical stimuli that cells are subjected to (Fig. 4b). This method is particularly suitable for in vitro tissue engineering, due to the high level of control over mechanical conditions achieved with various techniques (Huang and Niklason 2014). In the context of in situ tissue engineering, variations in scaffold geometry and material properties may be adopted to alter the hemodynamic loads presented to the cells in vivo (Loerakker et al. 2013; Wu et al. 2020; Tarrahi et al. 2020). For clinical translation, this indirect method may be preferred over direct manipulation from a regulatory perspective, as it does not require any active biological materials. Nevertheless, before such methods can successfully be applied in CVTE, some challenges might need to be addressed first. So far, many studies have investigated isolated signaling pathways or a limited selection of signaling pathways. To account for a more comprehensive influence of cell–cell signaling, it may be necessary to understand complex mechano-regulated signaling networks and crosstalk between different pathways. This requires increased research efforts in this area, for example to uncover how mechanical stimuli affect multiple pathways simultaneously by regulating certain shared downstream processes.

The consideration of mechano-regulated cell–cell signaling pathways in future CVTE studies will increase the already large number of variables in CVTE, which means that relying on experimental trial-and-error approaches alone quickly becomes impractical. There is, therefore, a clear need for a more systematic approach that enables efficient optimization of scaffold designs and CVTE protocols to identify the most promising combinations of parameters. We stress that computational modeling should be adopted in CVTE to complement the existing range of experimental strategies. Computational models have already been developed to enable the optimization of scaffold properties for tissue engineering of both blood vessels (Miller et al. 2015; Szafron et al. 2019) and heart valves (Loerakker et al. 2013; Emmert et al. 2018). Moreover, computational models have been successfully adopted to predict tissue G&R (Sect. 3.4) and unravel some of the complexities of cell–cell signaling pathways (Sect. 5.4). Combining these two types of models represents a coupling between biomechanics and systems biology and is an attractive opportunity for future studies, as it might enable the transition from a largely phenomenological description of tissue G&R to a more mechanistic one, motivated by cell–cell signaling pathways (Fig. 4a). The resulting framework would thus be able to both predict tissue G&R and describe some of the underlying mechanisms, thereby enabling the simulation of the signaling manipulations discussed in the previous paragraphs (Fig. 4b). This would at least partly answer the need for more biologically motivated G&R models that has previously been recognized (Miller et al. 2015; Szafron et al. 2018; Irons and Humphrey 2020; Irons et al. 2021). A compelling recent example is the study by Irons et al. (2021), in which a logic-based signaling model was coupled to a constrained mixture model for G&R which enables the modeling of bi-directional feedback between cell–cell signaling and tissue mechanics to improve our understanding of the role of signaling in G&R. The current model has been applied to simulate tissue G&R in response to hypertension, but similar models could in the future be used to study targeted manipulations of signaling pathways in CVTE.

### Remaining challenges

To inform these biologically motivated computational models, there is a clear need for more quantitative experimental data on the interplay between mechanical stimuli and cell–cell signaling, and the role of this interplay in tissue G&R. These data would also be highly valuable to quantitatively validate many of the models which are currently only validated qualitatively. In addition, there is a lack of tools to study signaling in real time in complex physiological environments, limiting the availability of data. For example, many of the in vitro signaling studies that are discussed in Sects. 5.1, 5.2, and 5.3 have used relatively simple mechanical conditions and only considered a limited number of time points. There is therefore a need for more real-time experimental data on signaling between cells that are subjected to more complex mechanical stimuli, such as anisotropic stress and strain. These conditions have been shown to have an important effect on cell behavior (chapter 4) and are more representative of the in vivo conditions of cells.

Including such complex biological behavior in computational models will inherently increase the number of model parameters. This can result in a higher uncertainty and difficulties regarding parameter estimation and model validation, especially considering the limited availability of experimental data. It therefore calls for a careful balance between complexity and simplicity in computational models, which should be tailored to specific research questions. Moreover, more complex models are associated with higher computational costs, especially when more complex geometries such as heart valves are modeled. This may require the development of more efficient computational techniques. Examples of such techniques implemented in previous studies include an analytical approximation of a stress fiber remodeling law (Ristori et al. 2016) and homogenized (Cyron et al. 2016) or time-independent (Latorre and Humphrey 2018) versions of the constrained mixture model for tissue G&R.

Challenges that remain in the field of cardiovascular tissue engineering and limit clinical translation include a large variability in outcome, suboptimal tissue function and organization, and the question whether engineered tissues can grow and adapt to changing demands, as discussed in Sect. 3.3. Moreover, some fundamental questions, for example regarding how cells repopulate an implanted scaffold, remain to be answered. An additional factor that plays a vital role in tissue regeneration is inflammation, which has been shown to be related to several cell–cell signaling pathways in the cardiovascular system, such as Notch and TGF-β signaling (Bartekova et al. 2018; Li and Kong 2020). This suggests that it may be important to consider the influence of cell–cell signaling on the inflammatory processes in future tissue engineering studies.

### Conclusion

In conclusion, mechano-regulated cell–cell signaling pathways may be a crucial link to explain the interplay between mechanical cues, cell behavior, and tissue G&R. An increased understanding of these cell–cell signaling pathways may be leveraged to improve the tissue organization and associated function of tissue-engineered blood vessels and heart valves, for example by activating scaffold surfaces with signaling molecules to induce or inhibit signaling interactions. We therefore propose that mechano-regulated cell–cell signaling is an important factor to consider in future CVTE studies. Computational models incorporating both tissue G&R and mechano-regulated cell–cell signaling pathways can provide an efficient tool to understand and predict how the interplay between mechanical cues and cell–cell signaling gives rise to certain tissue organizations. This will allow us to identify promising sets of scaffold parameters and tissue engineering protocols to reduce the vast experimental search space for obtaining a functional organization of engineered cardiovascular tissues.

## Abbreviations

BMP: Bone morphogenic protein
CVTE: Cardiovascular tissue engineering
Dll: Delta-like ligand
EC: Endothelial cell
ECM: Extracellular matrix
G&R: Growth and remodeling
MMP: Matrix metalloproteinase
NICD: Notch intracellular domain
VEC: Valvular endothelial cell
VIC: Valvular interstitial cell
VSMC: Vascular smooth muscle cell
TEHV: Tissue-engineered heart valve
TEBV: Tissue-engineered blood vessel
TEM: Tissue-engineered matrix
TGF-β: Transforming growth factor-*β*
GAG: Glycosaminoglycan
α-SMA: *α*-Smooth muscle actin
